# Sodium-Glucose Cotransporter 2 Inhibitors vs Incretin-Based Drugs and Risk of Fractures for Type 2 Diabetes

**DOI:** 10.1001/jamanetworkopen.2023.35797

**Published:** 2023-09-26

**Authors:** Hwa Yeon Ko, Sungho Bea, Han Eol Jeong, Sohee Park, Young Min Cho, Sung Hye Kong, Ju-Young Shin

**Affiliations:** 1 School of Pharmacy, Sungkyunkwan University, Suwon, South Korea; 2 Department of Biohealth Regulatory Science, Sungkyunkwan University, Suwon, South Korea; 3 Research Department of Practice and Policy, School of Pharmacy, University College London, London, United Kingdom; 4 Department of Internal Medicine, Seoul National University College of Medicine, Seoul, South Korea; 5 Division of Endocrinology and Metabolism, Department of Internal Medicine, Seoul National University Hospital, Seoul, South Korea; 6 Department of Internal Medicine, Seoul National University Bundang Hospital, Seongnam, South Korea; 7 Department of Clinical Research Design & Evaluation, Samsung Advanced Institute for Health Sciences & Technology, Sungkyunkwan University, Seoul, South Korea

## Abstract

**Importance:**

Postmenopausal individuals with type 2 diabetes are susceptible to fractures due to the interaction of elevated blood glucose levels and a deficiency of the hormone estrogen. Despite continued concerns of fracture risks associated with sodium-glucose cotransporter 2 inhibitors (SGLT2i), existing evidence in this high-risk population is lacking.

**Objective:**

To assess the risk of fractures associated with SGLT2i vs incretin-based drugs of dipeptidyl-peptidase 4 inhibitors (DPP4i) and glucagon-like peptide 1 receptor agonists (GLP1RA), separately, in postmenopausal individuals with type 2 diabetes.

**Design, Setting, and Participants:**

This active-comparator, new-user cohort study used nationwide claims data of Korea and took place from January 1, 2013, to December 31, 2020. Postmenopausal individuals (aged ≥45 years) with type 2 diabetes were included.

**Exposures:**

New users of SGLT2i or comparator drugs.

**Main Outcomes and Measures:**

The primary outcome was overall fractures, comprising vertebral, hip, humerus, and distal radius fractures. Patients were followed up from the day after drug initiation until the earliest of outcome occurrence, drug discontinuation (90-day grace period) or switch, death, or end of the study period. After propensity score fine stratification, hazard ratios (HRs) with 95% CIs were estimated using weighted Cox models.

**Results:**

Among 37 530 (mean [SD] age, 60.6 [9.7] years) and 332 004 (mean [SD] age, 60.6 [9.9] years) new users of SGLT2i and DPP4i, respectively, a lower rate of incident overall fractures was presented with SGLT2i vs DPP4i (weighted HR, 0.78; 95% CI, 0.72-0.84). Among 111 835 (mean [SD] age, 61.4 [9.8] years) and 8177 (mean [SD] age, 61.1 [10.3] years) new users of SGLT2i and GLP1RA, respectively, no association with an increased risk of overall fractures was presented with SGLT2i vs GLP1RA (weighted HR, 0.92; 95% CI, 0.68-1.24). Results from several subgroup and sensitivity analyses presented consistent results from main analysis.

**Conclusions and relevance:**

This population-based cohort study suggests that SGLT2i was not associated with an increased rate of incident fractures compared with DPP4i and GLP1RA, separately, among postmenopausal individuals with type 2 diabetes.

## Key Points

**Question:**

Do sodium-glucose cotransporter 2 inhibitors (SGLT2i) increase the risk of fractures among postmenopausal individuals with type 2 diabetes?

**Findings:**

In this Korean nationwide cohort study among the population susceptible for bone fragility, SGLT2i was not associated with the increased risk of fractures compared with the incretin-based drugs of dipeptidyl-peptidase 4 inhibitors and glucagon-like peptide 1 receptor agonists, separately.

**Meaning:**

In this study, SGLT2i use was associated with either similar or lower risks of fractures than incretin-based drugs even in a population with high risk of fractures.

## Teaser

This cohort study assesses the risk of fractures associated with sodium-glucose cotransporter 2 inhibitors vs incretin-based drugs of dipeptidyl-peptidase 4 inhibitors and glucagon-like peptide 1 receptor agonists, separately, in postmenopausal individuals with type 2 diabetes.

## Introduction

Postmenopausal individuals are at high risk of fractures due to declining estrogen levels that disrupt the homeostasis of bone metabolism.^1^ If these individuals also have type 2 diabetes, risk of fractures could be further heightened given that type 2 diabetes itself is an independent risk factor for fractures^2,3,4^; it might be attributed to chronic hyperglycemia and accumulation of advanced glycation end products, subsequently leading to altered bone metabolism and skeletal fragility.^5^ Therefore, potential fracture risks should be carefully weighed when choosing an optimal pharmacologic regimen for glycemic control in this population.

Sodium-glucose cotransporter 2 inhibitors (SGLT2i) have demonstrated substantial cardio-kidney benefits across several pivotal randomized clinical trials,^6,7,8,9^ and accordingly their usage in clinical practice is increasing.^10^ However, due to its unique glucose-controlling mechanism through kidney proximal tubules, SGLT2i may also affect calcium and phosphate homeostasis to possibly pose harm to bone mineral density.^11^ In support, one particular landmark trial of SGLT2i^7^ reported significantly increased rate of incident fractures compared with placebo (hazard ratio [HR], 1.26; 95% CI, 1.04-1.52). However, recent meta-analyses of trials have found no significant associations between SGLT2i and fractures.^12,13,14^ While several observational studies on this clinical topic are present,^15,16,17,18,19^ none to our knowledge have specifically assessed the association between SGLT2i use and fracture risk in postmenopausal individuals.

Given the current knowledge gap, we aimed to evaluate whether the use of SGLT2i is associated with an increased risk of fractures in a population that carries a greater underlying risk of bone fragility. Accordingly, we conducted a nationwide, population-based cohort study by comparing SGLT2i with incretin-based drugs in 2 independent cohorts of (1) SGLT2i vs dipeptidyl peptidase 4 inhibitors (DPP4i) and (2) SGLT2i vs glucagon-like peptide 1 receptor agonists (GLP1RA).

## Methods

### Data Source

We used health administrative claims data (January 1, 2013, to December 31, 2020) obtained from the National Health Insurance Service, a single provider for health insurance in South Korea. The national health insurance database contains health insurance claims data for roughly 97% of the entire Korean population (>50 million). Sociodemographic variables such as age, sex, residence, income level, eligibility status, and health insurance types are included. Health care utilization information such as diagnosis, prescription, medical procedures, and health examinations records are also available. Diagnosis records are coded according to the *International Statistical Classification of Diseases and Related Health Problems, Tenth Revision*, and drugs are coded based on a domestic drug chemical code that is mapped to the Anatomical Therapeutic Chemical classification of the World Health Organization. This study was approved by the institutional review board of Sungkyunkwan University, where requirement of informed consent was waived as this study used anonymized administrative data. This study followed the Strengthening the Reporting of Observational Studies in Epidemiology (STROBE) reporting guideline.^20^

### Study Population

We selected 2 active comparators for comparison with the SGLT2i and constructed 2 separate cohorts accordingly. Each cohort was constructed independently with patients with type 2 diabetes (*International Statistical Classification of Diseases and Related Health Problems, Tenth Revision* codes E11-E14), and all female individuals 45 years or older were included. We defined age of 45 years as a criterion for menopause based on the postmenopausal individuals definition of the Canagliflozin Cardiovascular Assessment Study (CANVAS) trial. Initially, we identified all individuals who initiated a SGLT2i or a comparator incretin-based drug (DPP4i or GLP1RA for each cohort) during the study period. The index date was defined as the date of the first prescription of either SGLT2i or a comparator incretin-based drug, whichever came first, from September 1, 2014 (ie, first date of SGLT2i reimbursement in Korea), to December 31, 2020; the forementioned drug classes were reimbursed exclusively for type 2 diabetes during this study period of interest. Patients whose date of the first prescription of either SGLT2i or a comparator drug was before September 1, 2014, were excluded. In this way, SGLT2i initiators who had previously used the comparator drug were excluded, while patients initiating comparator drug were restricted to those without a prior history of SGLT2i use.

Of these eligible individuals, we excluded those with severe kidney impairment (eg, end-stage kidney disease or received dialysis) within the year prior to the index date to consider for contraindication to SGLT2i. Since recurrences of fractures may not be independent, we further excluded patients who had records of fractures within the year prior to the index date. We also excluded patients with history of intensive care unit admission or diagnosis of cancer within the year prior to the index date. Last, we excluded patients who initiated both SGLT2i and a comparator incretin-based drug on the same date to avoid exposure misclassification (eFigures 1, 2, and 5 in Supplement 1).

### Exposure and Follow-Up

The drug of interest was SGLT2i (dapagliflozin, empagliflozin, ipragliflozin, or ertugliflozin). In each cohort, we chose DPP4i (alogliptin, evogliptin, gemigliptin, linagliptin, saxagliptin, sitagliptin, teneligliptin, or vildagliptin) and GLP1RA (albiglutide, dulaglutide, exenatide, or lixisenatide) as the active comparator with SGLT2i as they share the same line of therapy with SGLT2i in type 2 diabetes (ie, second- or third-line antihyperglycemic drug). Sulfonylureas and thiazolidinediones were not selected as appropriate active comparators with SGLT2i given their possible nonneutral effects on fractures.

Applying an as-treated approach, patients were followed up from the index date until the earliest of outcome occurrence, treatment change (discontinuation, switching to, or adding the comparator drug), death, or end of the study period (December 31, 2020). We introduced a 90-day grace period to determine treatment discontinuation; thus, patients were considered as exposed until 90 days after the end of days’ supply.

### Outcome Definition

The primary outcome was overall fractures, which comprised vertebral, hip, humerus, and distal radius fractures. Secondary outcomes were the individual components of the primary composite outcome. All outcomes were identified through diagnosis codes in the primary and secondary position, which have been previously reported to have a sensitivity of 63% to 93% and a positive predictive value of 60% to 97%.^21^ To further increase the validity of the outcomes, domestic procedure codes related to conservative therapy or procedure of each fracture sites were incorporated in sensitivity analyses (eg, stringent operational definition) (eTable 14 in Supplement 1).^22,23,24,25,26,27^

### Covariates

We assessed calendar year and age (45-65, >65 years) on the index date and the number of antidiabetic medications (other than SGLT2i, incretin-based drugs) prescribed in the year prior to index date. We also defined levels of diabetes treatment into 3 levels depending on the number of antidiabetic medication (excluding the study drugs of interest), prescribed in the year preceding the index date: level 1, as taking none or only 1 class of antidiabetic medication other than insulin, level 2, as taking 2 or more different classes of antidiabetic medication without insulin, and level 3, as taking insulin with or without other classes of antidiabetic medication. Moreover, as proxies for health-seeking behavior, the number of outpatient visits and the number of hospitalizations were assessed within the year prior to the index date. Clinical characteristics including the Charlson Comorbidity Index, comorbidities, and comedications, were also assessed within the year prior to the index date. Comorbidities of interest, identified using relevant diagnostic codes (eTable 15 in Supplement 1), were diabetes-related conditions (nephropathy, neuropathy, retinopathy, and hypoglycemia), asthma, cancer, chronic kidney disease, congestive heart failure, chronic obstructive pulmonary disease, dementia, epilepsy, gout, hyperlipidemia, hypertension, ischemic heart disease, liver cirrhosis, osteoarthritis, osteoporosis, rheumatoid arthritis, stroke, and thyroid disease. Use of fall-related medications (angiotensin-converting enzyme inhibitors, angiotensin receptor blockers, anticholinergics, benzodiazepines, β-blockers, calcium channel blockers, diuretics, nitrates, opioids, sedative hypnotics, tricyclic antidepressant, and typical antipsychotics), osteoporosis medications (bisphosphonates, calcium/vitamin D, parathyroid hormone/calcitonin, receptor activator of nuclear factors kappa-B ligand inhibitors, and selective estrogen receptor modulators), and other comedications (anticoagulants, anticonvulsants, antidepressants, aromatase inhibitors, oral corticosteroids, hormone replacement therapy, immunosuppressants, nonsteroidal anti-inflammatory drugs, platelet inhibitors, proton-pump inhibitors, and statins) were also evaluated (eTable 16 in Supplement 1). Laboratory test results (missing rates, 27.9%-57.6%) from biennial health examination data were assessed within 3 years prior to the index date and included in the propensity score model for sensitivity analysis (eTable 10 in Supplement 1). Results of bone mineral density test using dual-energy x-ray absorptiometry were also assessed within 3 years prior to the index date among a subset of population.

### Statistical Analysis

Descriptive statistics were used to compare patients’ baseline characteristics in each cohort. Continuous variables were presented as means and SDs, and categorial variables were summarized as frequency and proportions. We used the propensity score fine stratification weighting method^28^ to control for potential confounding within each cohort (SGLT2i vs DPP4i; SGLT2i vs GLP1RA). Multivariable logistic regression model was used to estimate the predicted probability of initiating SGLT2i vs incretin-based drugs (DPP4i or GLP1RA) given all baseline covariates mentioned above. Patients from nonoverlapping regions of propensity score distributions were trimmed to focus the estimation of treatment effects in a population with clinical equipoise. We created 50 strata based on the distribution of propensity score in the SGLT2i group. Within each stratum, patients exposed to SGLT2i were assigned a weight of 1, while patients exposed to comparator drugs were weighted in proportion to the number of SGLT2i-exposed patients in the stratum into which they fell; this would then measure the mean treatment effect among treated patients. Absolute standardized differences larger than 0.1 were considered as significant covariate imbalance between treatment groups. We also estimated postweighting C statistics that serves as a measure of balance (0.5 denotes balance) in aggregate over all included covariates. Weighted incidence rates based on the Poisson distribution were estimated for study outcomes by dividing the number of weighted events by the total number of weighted 100 patients-years at risk. Weighted Kaplan-Meier plots were used to visualize cumulative incidence of the primary outcome (eFigure 3 in Supplement 1). Weighted Cox proportional hazard models were used to estimate weighted HRs and 95% CIs for the risk of fractures associated with SGLT2i vs incretin-based drugs.

Subgroup analyses were conducted to test for potential effect modification. First, we stratified on age (45-60, 61-75, >75 years) to identify whether higher age cutoffs for menopause modify effect estimates. Second, we also stratified on history of osteoporosis or use of osteoporosis medications, history of osteoarthritis, history of neurological dysfunction, and prior use of drugs that increase fracture risks (thiazolidinediones, proton pump inhibitor, systemic corticosteroid, selective serotonin reuptake inhibitor). Third, we examined the effect of individual SGLT2i associated with fractures. In all subgroup analyses, propensity score were reestimated. *P* values for interaction less than .05 were used to denote a significant heterogeneity among subgroups.

Range of sensitivity analyses were conducted for the primary outcome to assess the robustness of main analysis. First, we repeated the main analysis among patients with a history of hysterectomy or ovarian resection (eg, conditions that lead to surgical menopause) any time before the index date or patients with a history of hormone replacement therapy any time before the index date to increase the validity of age-based definition. Second, we repeated the main analysis including laboratory test results as covariates for the propensity score model. Third, we extended the assessment period for baseline comorbidities to include any time before the index date. Fourth, we varied the length of the grace period to 30- and 60-day periods to consider potential exposure misclassifications. Fifth, we adopted a more stringent outcome definition by using domestic procedural codes related to conservative therapy or procedures of each fracture sites to minimize outcome misclassification. Sixth, to indirectly assess the presence of potential unmeasured confounding, we used 2 control outcomes: (1) herpes zoster virus infection as a negative control outcome, where a null association was expected and (2) hospitalization for heart failure as a positive control outcome, where a lower risk with SGLT2i was anticipated. These analyses were based on the same cohort as the one used in the main analysis but involved excluding patients diagnosed with herpes zoster virus infection or heart failure a year prior to the index date. Finally, we repeated the main analysis among a subset of participants with bone mineral density test results, including the results as covariates for the propensity score model. All statistical analyses were conducted with SAS software, version 9.4 (SAS Institute Inc).

## Results

A total of 369 570 patients were selected for the first cohort: 37 532 patients initiating SGLT2i (mean [SD] age, 60.6 [9.7] years) and 332 038 patients initiating DPP4i (mean [SD] age, 66.0 [11] years) (eFigure 1 in Supplement 1). Regarding the second cohort, a total of 121 803 patients were selected: 113 622 patients initiating SGLT2i (mean [SD] age, 61.4 [9.8] years) and 8181 patients initiating GLP1RA (mean [SD] age, 62.5 [10.2] years) (eFigure 2 in Supplement 1). Patients in the second cohort presented a higher prevalence of diabetes-related conditions, insulin usage, and a greater number of diabetic medications taken, indicating a generally more severe diabetic profile compared with the first cohort (eTable 1 in Supplement 1).

After propensity score weighting and trimming, the first cohort had 37 530 patients initiating SGLT2i and 332 004 patients initiating DPP4i, whereas the second cohort had 111 835 patients initiating SGLT2i and 8177 patients initiating GLP1RA. In both cohorts, the exposure and comparator groups were well balanced, with absolute standardized differences for all baseline covariates less than 0.10 after propensity score weighting (Table 1). The overall balance was further confirmed using the postweighting C statistics (0.504 for the first cohort, 0.567 for the second cohort) (eFigure 5 in Supplement 1).

**Table 1.** Baseline Characteristics of Patients Who Received SGLT2i or Comparator Drugs After Weighting by Propensity Score Fine Stratification

Baseline characteristic	Patients, No. (%)					
	SGLT2i vs DPP4i			SGLT2i vs GLP1RA		
	SGLT2i (n = 37 530)	DPP4i (n = 332 004)	ASD	SGLT2i (n = 111 835)	GLP1 RA (n = 8177)	ASD
Cohort entry year						
2014	1232 (3.3)	10 957 (3.3)	0.001	2499 (2.2)	166 (2)	0.014
2015	3921 (10.4)	34 628 (10.4)	0.001	9419 (8.4)	714 (8.7)	0.011
2016	5220 (13.9)	46 175 (13.9)	<0.001	16 845 (15.1)	1225 (15)	0.002
2017	6310 (16.8)	55 960 (16.9)	0.001	19 557 (17.5)	1545 (18.9)	0.036
2018	5815 (15.5)	51 432 (15.5)	<0.001	18 336 (16.4)	1286 (15.7)	0.018
2019	7414 (19.8)	65 512 (19.7)	0.001	23 212 (20.8)	1663 (20.3)	0.01
2020	7618 (20.3)	67 340 (20.3)	<0.001	21 967 (19.6)	1579 (19.3)	0.008
Age, mean (SD), y	60.6 (9.7)	60.6 (9.9)	0.001	61.4 (9.8)	61.1 (10.3)	0.039
Age group						
45-65	26 760 (71.3)	237 418 (71.5)	0.001	75 939 (67.9)	5613 (68.6)	0.016
>65	10 770 (28.7)	94 626 (28.5)		35 896 (32.1)	2564 (31.4)	
Diabetic medications						
Alpha-glucosidase inhibitors	1141 (3)	10 117 (3)	<0.001	3141 (2.8)	259 (3.2)	0.021
GLP1RA	126 (0.3)	868 (0.3)	0.014	NA	NA	NA
DPP4i	NA	NA	NA	68 426 (61.2)	4864 (59.5)	0.035
Insulin	3258 (8.7)	28 607 (8.6)	0.002	15 717 (14.1)	1265 (15.5)	0.04
Meglitinides	191 (0.5)	1681 (0.5)	<0.001	580 (0.5)	49 (0.6)	0.011
Metformin	21 188 (56.5)	187 378 (56.4)	<0.001	90 052 (80.5)	6372 (77.9)	0.064
Sulfonylureas	9388 (25)	83 027 (25)	<0.001	53 286 (47.6)	4034 (49.3)	0.034
Thiazolidinediones	2708 (7.2)	23 821 (7.2)	0.002	15 904 (14.2)	1264 (15.5)	0.035
No. of diabetic medications being taken						
0-1	26 469 (70.5)	234 486 (70.6)	0.002	29 365 (26.3)	2197 (26.9)	0.014
2-3	10 752 (28.6)	94 809 (28.6)	0.002	69 311 (62)	4851 (59.3)	0.054
≥4	309 (0.8)	2709 (0.8)	0.001	13 159 (11.8)	1129 (13.8)	0.061
Level of diabetes treatment^a^						
1	25 926 (69.1)	229 636 (69.2)	0.002	28 629 (25.6)	2144 (26.2)	0.014
2	8346 (22.2)	73 761 (22.2)	0.001	67 489 (60.3)	4767 (58.3)	0.042
3	3258 (8.7)	28 607 (8.6)	0.002	15 717 (14.1)	1265 (15.5)	0.04
Diabetes related conditions						
Diabetic nephropathy	1148 (3.1)	10 107 (3)	0.001	5811 (5.2)	401 (4.9)	0.014
Diabetic neuropathy	4595 (12.2)	40 551 (12.2)	0.001	20 639 (18.5)	1543 (18.9)	0.011
Diabetic retinopathy	5730 (15.3)	50 450 (15.2)	0.002	25 723 (23)	1818 (22.2)	0.018
Hypoglycemia	130 (0.3)	1154 (0.3)	<0.001	543 (0.5)	34 (0.4)	0.01
Charlson Comorbidity Index						
0	19 584 (52.2)	173 208 (52.2)	<0.001	49 318 (44.1)	3450 (42.2)	0.039
1	10 586 (28.2)	93 614 (28.2)	<0.001	40 709 (36.4)	3116 (38.1)	0.035
2	4385 (11.7)	38 826 (11.7)	<0.001	10 796 (9.7)	782 (9.6)	0.003
≥3	2975 (7.9)	26 356 (7.9)	<0.001	11 012 (9.8)	830 (10.2)	0.01
No. of outpatients visits						
0-2	1038 (2.8)	9264 (2.8)	0.002	1451 (1.3)	177 (2.2)	0.067
3-5	1738 (4.6)	15 415 (4.6)	0.001	3034 (2.7)	231 (2.8)	0.007
≥6	34 754 (92.6)	307 325 (92.6)	0.001	107 350 (96)	7769 (95)	0.047
No. of hospitalizations						
0	30 118 (80.3)	266 471 (80.3)	<0.001	85 439 (76.4)	5882 (71.9)	0.102
1-2	6815 (18.2)	60 230 (18.1)	<0.001	23 680 (21.2)	2060 (25.2)	0.095
≥3	597 (1.6)	5303 (1.6)	0.001	2716 (2.4)	235 (2.9)	0.028
Comorbidities						
Asthma	3249 (8.7)	28 665 (8.6)	0.001	9514 (8.5)	745 (9.1)	0.021
Chronic kidney disease	201 (0.5)	1830 (0.6)	0.002	1160 (1)	97 (1.2)	0.014
Congestive heart failure	1481 (3.9)	13 012 (3.9)	0.001	4769 (4.3)	310 (3.8)	0.024
COPD	2036 (5.4)	18 027 (5.4)	<0.001	6164 (5.5)	528 (6.5)	0.04
Dementia	1001 (2.7)	8994 (2.7)	0.003	3701 (3.3)	292 (3.6)	0.014
Epilepsy	227 (0.6)	2011 (0.6)	<0.001	685 (0.6)	54 (0.7)	0.006
Gout	260 (0.7)	2314 (0.7)	0.001	823 (0.7)	54 (0.7)	0.009
Hyperlipidemia	16 975 (45.2)	149 714 (45.1)	0.003	52 526 (47)	3772 (46.1)	0.017
Hypertension	20 253 (54)	178 733 (53.8)	0.003	60 294 (53.9)	4389 (53.7)	0.005
Ischemic heart disease	3273 (8.7)	28 763 (8.7)	0.002	11 078 (9.9)	758 (9.3)	0.022
Liver cirrhosis	144 (0.4)	1289 (0.4)	0.001	635 (0.6)	48 (0.6)	0.003
Osteoarthritis	12 546 (33.4)	110 917 (33.4)	<0.001	38 494 (34.4)	2830 (34.6)	0.004
Osteoporosis	3268 (8.7)	28 820 (8.7)	0.001	9972 (8.9)	682 (8.3)	0.02
Parkinson disease	129 (0.3)	1165 (0.4)	0.001	497 (0.4)	45 (0.6)	0.015
Rheumatoid arthritis	778 (2.1)	6901 (2.1)	<0.001	2363 (2.1)	178 (2.2)	0.004
Stroke	1068 (2.8)	9506 (2.9)	0.001	3902 (3.5)	289 (3.5)	0.003
Thyroid disease	2951 (7.9)	25 947 (7.8)	0.002	8143 (7.3)	634 (7.8)	0.018
Fall-related medications						
ACE inhibitors or ARBs	19 035 (50.7)	167 677 (50.5)	0.004	60 846 (54.4)	4471 (54.7)	0.005
Anticholinergics	30 173 (80.4)	266 797 (80.4)	0.001	91 291 (81.6)	6711 (82.1)	0.011
Benzodiazepines	13 565 (36.1)	119 957 (36.1)	<0.001	41 469 (37.1)	3061 (37.4)	0.007
β-Blockers	1848 (4.9)	16 290 (4.9)	0.001	5512 (4.9)	398 (4.9)	0.003
Calcium channel blockers	14 617 (38.9)	128 939 (38.8)	0.002	43 791 (39.2)	3177 (38.9)	0.006
Diuretics	9571 (25.5)	84 374 (25.4)	0.002	28 869 (25.8)	2205 (27)	0.026
Nitrates	470 (1.3)	4120 (1.2)	0.001	1643 (1.5)	86 (1.1)	0.037
Opioids	2875 (7.7)	25 364 (7.6)	0.001	9612 (8.6)	914 (11.2)	0.087
Sedative hypnotics	3976 (10.6)	35 143 (10.6)	<0.001	12 507 (11.2)	937 (11.5)	0.009
Tricyclic antidepressant	2633 (7)	23 285 (7)	<0.001	9056 (8.1)	716 (8.8)	0.024
Typical antipsychotics	1227 (3.3)	10 837 (3.3)	<0.001	3875 (3.5)	355 (4.3)	0.045
Osteoporosis medications						
Bisphosphonates	1737 (4.6)	15 346 (4.6)	<0.001	5529 (4.9)	320 (3.9)	0.05
Calcium/vitamin D	2903 (7.7)	25 547 (7.7)	0.002	9346 (8.4)	671 (8.2)	0.005
Parathyroid hormone/calcitonin	108 (0.3)	964 (0.3)	<0.001	384 (0.3)	19 (0.2)	0.021
RANKL inhibitors	134 (0.4)	1181 (0.4)	<0.001	470 (0.4)	32 (0.4)	0.004
SERMs	440 (1.2)	3882 (1.2)	<0.001	1348 (1.2)	78 (1)	0.024
Comedications						
Anticoagulants	1287 (3.4)	11 337 (3.4)	0.001	4617 (4.1)	398 (4.9)	0.036
Anticonvulsants	4301 (11.5)	37 984 (11.4)	0.001	17 293 (15.5)	1358 (16.6)	0.031
Antidepressants	2806 (7.5)	24 661 (7.4)	0.002	9172 (8.2)	815 (10)	0.061
Aromatase inhibitors	2 (0)	16 (0)	0.001	0 (0)	0 (0)	NA
Oral corticosteroids	14 825 (39.5)	131 199 (39.5)	<0.001	41 631 (37.2)	3136 (38.4)	0.023
Hormone replacement therapy	2180 (5.8)	19 339 (5.8)	0.001	6300 (5.6)	494 (6)	0.018
Immunosuppressants	113 (0.3)	1016 (0.3)	0.001	382 (0.3)	49 (0.6)	0.038
NSAIDs	25 971 (69.2)	229 672 (69.2)	0.001	79 021 (70.7)	5773 (70.6)	0.001
Platelet inhibitors	23 792 (63.4)	210 321 (63.3)	0.001	75 427 (67.4)	5511 (67.4)	0.001
Proton-pump inhibitors	14 685 (39.1)	129 908 (39.1)	<0.001	46 368 (41.5)	3480 (42.6)	0.022
Statins	22 388 (59.7)	197 423 (59.5)	0.004	79 510 (71.1)	5537 (67.7)	0.073

Abbreviations: ACE, angiotensin-converting enzyme; ARBs, angiotensin receptor blockers; ASD, absolute standardized difference; COPD, chronic obstructive pulmonary disorder; DPP4i, dipeptidyl peptidase 4 inhibitors; GLP1RA, glucagon-like peptide 1 receptor agonists; NA, not applicable; NSAIDs, nonsteroidal anti-inflammatory drugs; RANKL, receptor activator of nuclear factors kappa B ligand; SERMs, selective estrogen receptor modulator; SGLT2i, sodium glucose cotransporter 2 inhibitors.

^a^ Defined depending on the number of antidiabetic medication (excluding the study drugs of interest), prescribed in the year preceding the index date: level 1, taking none or 1 class of antidiabetic medication other than insulin; level 2, taking 2 or more different classes of antidiabetic medication without insulin; and level 3, taking insulin with or without other classes of antidiabetic medication.

During a mean (SD) follow-up of 1.45 (1.42) years and 2.09 (1.79) years, users of SGLT2i and DPP4i yielded a weighted incidence of 1.41 and 1.81 events per 100 person-years for the overall fractures, respectively, corresponding to a 22% lower rate of incident overall fractures with SGLT2i vs DPP4i (weighted HR, 0.78; 95% CI, 0.72-0.84). Meanwhile, over a mean (SD) follow-up of 1.43 (1.3) years and 0.82 (0.85) years, the weighted incidence of overall fractures was 1.67 per 100 person-years for SGLT2i and 1.92 per 100 person-years for GLP1RA. Use of SGLT2i, compared with GLP1RA, was not associated with overall fractures (weighted HR, 0.92; 95% CI, 0.68-1.24) (Table 2; eTables 8 and 9 and eFigure 4 in Supplement 1). Similar trends were found for the secondary outcomes across both cohorts, where SGLT2i use did not increase the risk of fractures, regardless of the comparator incretin-based drug (eTables 2 and 5 in Supplement 1).

**Table 2.** Association Between the Use of SGLT2i and the Risk of Overall Fractures Among Weighted Populations

Exposure	No. of patients^a^	No. of Events	Person-years	Weighted IR (95% CI)^b^	Weighted HR (95% CI)
SGLT2i vs DPP4i					
SGLT2i	37 530	768	54 619	1.41 (1.31-1.51)	0.78 (0.72-0.84)
DPP4i	332 004	18 800	693 818	1.81 (1.78-1.85)	1.00 [Reference]
SGLT2i vs GLP1RA					
SGLT2i	111 835	2723	164 967	1.67 (1.60-1.73)	0.92 (0.68-1.24)
GLP1RA	8177	156	6737	1.92 (1.59-2.30)	1.00 [Reference]

Abbreviations: DPP4i, dipeptidyl peptidase 4 inhibitors; GLP1RA, glucagon-like peptide 1 receptor agonists; HR, hazard ratio; IR, incidence rate; SGLT2i, sodium-glucose cotransporter 2 inhibitors.

^a^ Patients remained after trimming patients in the nonoverlapping regions of the propensity score distributions.

^b^ Per 100 person-years.

**Figure 1.**
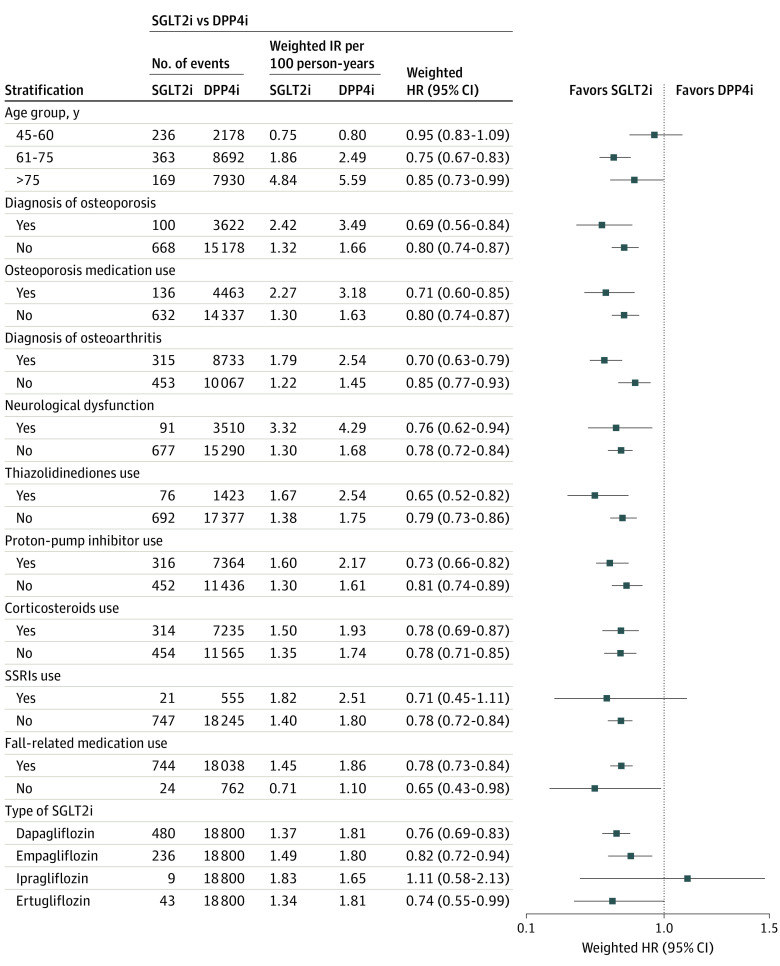
Results of Subgroup Analyses With Events for the Association Between the Use of Sodium-Glucose Cotransporter 2 Inhibitors (SGLT2i) vs Dipeptidyl Peptidase 4 Inhibitors (DPP4i) and the Risk of Overall Fractures HR indicates hazard ratio; IR, incidence rate; SSRI, selective serotonin reuptake inhibitor.

**Figure 2.**
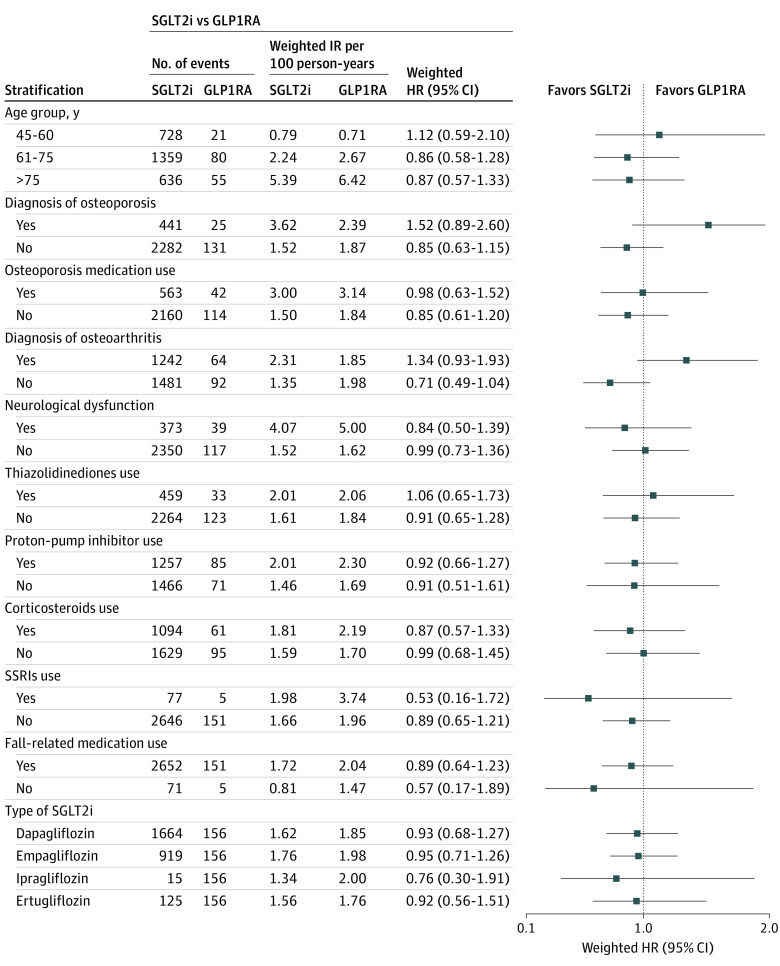
Results of Subgroup Analyses With Events for the Association Between the Use of Sodium-Glucose Cotransporter 2 Inhibitors (SGLT2i) vs Glucagon-Like Peptide 1 Receptor Agonists (GLP1RA) and the Risk of Overall Fractures HR indicates hazard ratio; IR, incidence rate; SSRI, selective serotonin reuptake inhibitor.

Results of subgroup analyses found no significant effect modification by age and prespecified covariates known to increase fracture risks, except for history of osteoarthritis; patients with a history of osteoarthritis (HR, 0.70; 95% CI, 0.63-0.79) had a lower rate of incident fractures with SGLT2i vs DPP4i than their counterpart (HR, 0.85; 95% CI, 0.77-0.93; *P* for interaction = .02) (Figure 1 and Figure 2; eTable 3 in Supplement 1). On the contrary, patients with a history of osteoarthritis (HR, 1.34; 95% CI, 0.93-1.93) had a significantly higher rate of incident fractures with SGLT2i vs GLP1RA, while their counterpart had a significantly lower rate of outcome (HR, 0.71; 95% CI, 0.49-1.04; *P* for interaction = .03) (eTable 6 in Supplement 1). All ingredients of SGLT2i-generated HR estimates below the null, although ipragliflozin had very wide CIs likely due to the relatively low numbers of events.

**Figure 3.**
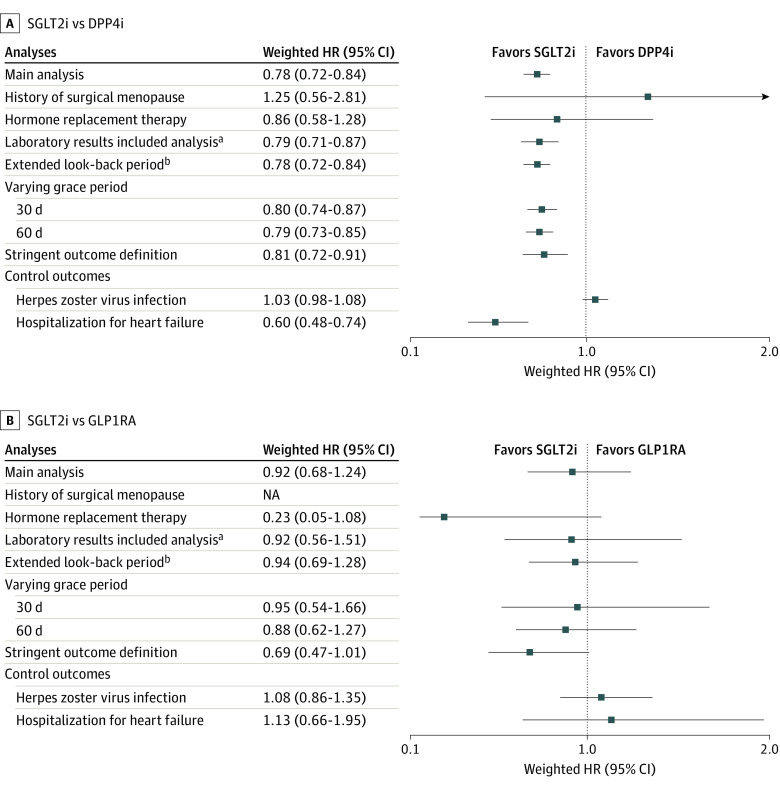
Results of Sensitivity Analyses for the Association Between the Use of Sodium-Glucose Cotransporter 2 Inhibitors (SGLT2i) vs Dipeptidyl Peptidase 4 Inhibitors (DPP4i) and the Use of SGLT2i vs Glucagon-Like Peptide 1 Receptor Agonists (GLP1RA) and the Risk of Overall Fractures HR indicates hazard ratio; NA, not applicable. ^a^Laboratory test results were assessed within 3 years prior to the index date and the most recent value was included. Included laboratory variables comprised waist circumference, body mass index, fasting blood glucose, systolic blood pressure, diastolic blood pressure, total cholesterol, low-density lipoprotein cholesterol, high-density lipoprotein cholesterol, triglycerides, serum creatinine, estimated glomerular filtration rate, aspartate aminotransferase level, alanine aminotransferase level, gamma glutamyl transferase level, and smoking behavior, which presented varying missing rates of 27.9% to 60.5% for each variable. ^b^Assessment period for baseline comorbidities was extended to include any time before the index date.

Across both cohorts, results of various sensitivity analyses were generally consistent, including the analysis that restricted to patients with a history of surgical menopause or hormone replacement therapy, the analysis that included laboratory results as covariates or extended comorbidity assessment period, and the analysis that used a stringent outcome definition. Moreover, null associations were consistently observed for the negative control outcomes, whereas a significantly lower risk was observed for the positive control outcome, which is in line with current knowledge (Figure 3; eTables 4, 7, 11-13 in Supplement 1).^29^

## Discussion

In this large-scale, nationwide cohort study that constructed 2 independent active comparator, new user cohorts, we found no increased risk of fractures among postmenopausal individuals with type 2 diabetes that initiated and continued treatment with SGLT2i vs DPP4i or GLP1RA. These findings were consistent across several subgroup and sensitivity analyses.

To our knowledge, no study, trial or observational, has specifically examined the risk of fractures with SGLT2i in postmenopausal individuals with type 2 diabetes, making it difficult to make any formal comparisons with existing data. Yet, our study was in agreement with a few studies that provide grounds for indirect comparisons. Several randomized clinical trials^8,30^ of SGLT2i other than canagliflozin and meta-analyses after the CANVAS trial have found little to no evidence of fracture risks.^31,32^ To date, 2 observational studies have assessed the risk of fractures associated with SGLT2i among patients with type 2 diabetes. The first cohort study, which used US Medicare claims data,^18^ found no association with an increased risk of fractures with SGLT2i among female individuals 66 years or older compared with DPP4i (HR, 0.88; 95% CI, 0.68-1.12) and GLP1RA (HR, 1.10; 95% CI, 0.83-1.45). Another cohort study using the UK Clinical Practice Research Datalink^15^ reported similar findings of no association with an increased risk of fractures among female individuals 40 years or older using SGLT2i vs DPP4i (HR, 0.88; 95% CI, 0.67-1.17). While these previous cohort studies did not investigate postmenopausal individuals specifically, their age cutoffs among female individuals support our findings of no increased risk of fractures with SGLT2i. Nevertheless, more studies in this population are needed to corroborate our findings.

Biologically, SGLT2i may potentially disrupt calcium and phosphate homeostasis in serum by increasing proximal tubular reabsorption of phosphate, which might have detrimental effects on bone and lead to skeletal fragility.^11,33^ This risk is expected to be further exacerbated in postmenopausal individuals given their decreased levels of estrogen hormones and consequent disruption of bone metabolism homeostasis. Moreover, SGLT2i have demonstrated an indirect elevation of bone turnover through weight loss. Increased levels of osteocalcin and C-terminal telopeptide of type I collagen, established indicators of bone turnover, have been detected subsequent to adipose tissue reduction, potentially exerting a detrimental influence on bone mineral density.^34^ Despite such biological plausibility, our results found a modest potential protective effect of SGLT2i against overall fractures, which may be explained by the role of SGLT2i in the trajectory of impaired bone metabolism caused by the accumulation of advanced glycation end products in bone collagen fibers in advanced diabetes.^35,36^ Persistent hyperglycemia accelerates the formation of advanced glycation end products such as pentosidine and N-carboxymethyl-lysine, which contributes to oxidative stress in the bone microenvironment to promote collagen cross-linking, making bone stiffer and more susceptible to fractures.^37,38,39^ Several experimental studies have reported that SGLT2i exhibit inhibitory effects on the advanced glycation end products–receptor for advanced glycation end products signaling pathway via ameliorating glucose toxic effects,^41^ and thereby having a protective effect against fractures. Nevertheless, there remains much to be understood regarding the exact biological mechanism SGLT2i, DPP4i, and GLP1RA have in bone metabolism homeostasis. Thus, further research is needed to better elucidate this observation.

### Strengths and Limitations

Our study has several strengths. To our knowledge, this is the first study to assess the risk of fractures associated with commonly used antidiabetic regimens in routine practice among postmenopausal individuals with type 2 diabetes. Second, we constructed 2 separate cohorts to provide comprehensive comparative safety evidence on female diabetic patients using SGLT2i vs DPP4i and SGLTI2i vs GLP1RA, or medications that share treatment stage in type 2 diabetes management, which are therefore clinically meaningful. Third, by conducting several subgroup analyses that accounted for various risk factors for fractures, we validated the robustness of our main finding and established the safety of SGLT2i for the majority of patients, although those with osteoarthritis warrant further attention. Fourth, we minimized the potential for outcome misclassification by applying an algorithm of operational definition for fractures that included procedure codes stratified by skeletal sites, which have been previously validated. Fifth, we used propensity score fine stratification weights, allowing us to preserve most observations in the analysis and produce more precise effect estimates.^28^

This study has some limitations. First, residual confounding due to unmeasured covariates cannot be ruled out, although we adjusted for various measurable covariates; in particular, we assessed the severity of diabetes and multiple baseline risk factors of fracture. Second, the number of patients using GLP1RA was low, which may limit the generalizability of the SGLT2i vs GLP1RA comparison. Thus, further studies using more recent data are needed to better estimate this association. Third, since all study participants were Korean, our findings may not be generalized to other populations. Last, the lack of a standardized definition of postmenopausal status is a limitation in epidemiological studies using claims data. Nevertheless, several validation studies have shown that using age as a proxy was not significantly different from using other, more complex definitions.^42^ To further increase the validity, we used other measures (eg, surgery and hormone replacement therapy) that can complement the age-based definition as a sensitivity analysis, which revealed consistent findings. Also, in a subgroup analysis among patients 61 years or older, who were expected to be almost certain postmenopause, no trend of an increased risk of fracture with SGLT2i was observed.

## Conclusions

The use of SGLT2i was not associated with an increased risk of overall fractures among postmenopausal patients with type 2 diabetes. This result remained consistent irrespective of the particular incretin-based drug as an active comparator. These findings indicate that SGLT2i has either similar or lower risks of fractures than incretin-based drugs even in a population at higher risk for fractures, providing reassurance to and helping health care professionals with their clinical decision-making.

## Notes

Supplement 1. **eFigure 1.** Flowchart of study participants selection **eFigure 2.** Flowchart of study participants selection **eFigure 3.** Cumulative incidence function plots for overall fractures among weighted populations **eFigure 4.** Overall study design **eFigure 5.** Propensity score distribution of before and after weighting in both cohorts **eTable 1.** Baseline characteristics of patients received SGLT2 inhibitors or comparator drugs, before weighting by propensity score fine stratification **eTable 2.** Association between the use of SGLT2 inhibitors and the risk of fractures compared with the use of DPP4 inhibitors **eTable 3.** Results of subgroup analyses with events, person-years, weighted IRs (per 100 person-years), weighted HRs with 95% CIs, and p for interaction for the association between the use of SGLT2 inhibitors vs DPP4 inhibitors and the risk of overall fractures **eTable 4.** Results of sensitivity analyses with events, person-years, weighted IRs (per 100 person-years), weighted HRs and 95% CIs for the association between the use of SGLT2 inhibitors vs DPP4 inhibitors and the risk of overall fractures **eTable 5.** Association between the use of SGLT2 inhibitors and the risk of fractures compared with the use of GLP-1 receptor agonists **eTable 6.** Results of subgroup analyses with events, person-years, weighted IRs (per 100 person-years), weighted HRs with 95% CIs, and p for interaction for the association between the use of SGLT2 inhibitors vs GLP-1 receptor agonists and the risk of overall fractures **eTable 7.** Results of sensitivity analyses with events, person-years, weighted IRs (per 100 person-years), weighted HRs and 95% CIs for the association between the use of SGLT2 inhibitors vs GLP-1 receptor agonists and the risk of overall fractures **eTable 8.** Reasons for the end of follow-up among weighted populations received SGLT2 inhibitors or DPP4 inhibitors **eTable 9.** Reasons for the end of follow-up among weighted populations received SGLT2 inhibitors or GLP-1 receptor agonists **eTable 10.** Laboratory test results and missing rates of patients receiving SGLT2 inhibitors or comparator drugs before propensity score based fine stratification **eTable 11.** Baseline characteristics after weighting by propensity score stratification in patients receiving SGLT2 inhibitors or comparator drugs with bone mineral density test results present **eTable 12.** Baseline characteristics before weighting by propensity score stratification in patients receiving SGLT2 inhibitors or comparator drugs with bone mineral density test results present **eTable 13.** Association between the use of SGLT2 inhibitors and the risk of overall fractures among weighted populations with bone mineral density test results present **eTable 14.** International Classification of Diseases-10 (ICD-10) and domestic procedure codes for identification of fractures **eTable 15.** International Classification of Diseases-10 (ICD-10) codes for identification of covariates **eTable 16.** Anatomical Therapeutic Chemical (ATC) classification codes for identification of covariates

Supplement 2.
Data Sharing Statement
