# Supplementary material for: Sodium-Glucose Cotransporter 2 Inhibitors vs Incretin-Based Drugs and Risk of Fractures for Type 2 Diabetes
Source: JAMA Netw Open. 2023 Sep 26;6(9):e2335797. doi: 10.1001/jamanetworkopen.2023.35797 (PMC10523172; doi:10.1001/jamanetworkopen.2023.35797)
Supplement: Supplement 1. — eFigure 1. Flowchart of study participants selection eFigure 2. Flowchart of study participants selection eFigure 3. Cumulative incidence function plots for overall fractures among weighted populations eFigure 4. Overall study design eFigure 5. Propensity score distribution of before and after weighting in both cohorts eTable 1. Baseline characteristics of patients received SGLT2 inhibitors or comparator drugs, before weighting by propensity score fine stratification eTable 2. Association between the use of SGLT2 inhibitors and the risk of fractures compared with the use of DPP4 inhibitors eTable 3. Results of subgroup analyses with events, person-years, weighted IRs (per 100 person-years), weighted HRs with 95% CIs, and p for interaction for the association between the use of SGLT2 inhibitors vs DPP4 inhibitors and the risk of overall fractures eTable 4. Results of sensitivity analyses with events, person-years, weighted IRs (per 100 person-years), weighted HRs and 95% CIs for the association between the use of SGLT2 inhibitors vs DPP4 inhibitors and the risk of overall fractures eTable 5. Association between the use of SGLT2 inhibitors and the risk of fractures compared with the use of GLP-1 receptor agonists eTable 6. Results of subgroup analyses with events, person-years, weighted IRs (per 100 person-years), weighted HRs with 95% CIs, and p for interaction for the association between the use of SGLT2 inhibitors vs GLP-1 receptor agonists and the risk of overall fractures eTable 7. Results of sensitivity analyses with events, person-years, weighted IRs (per 100 person-years), weighted HRs and 95% CIs for the association between the use of SGLT2 inhibitors vs GLP-1 receptor agonists and the risk of overall fractures eTable 8. Reasons for the end of follow-up among weighted populations received SGLT2 inhibitors or DPP4 inhibitors eTable 9. Reasons for the end of follow-up among weighted populations received SGLT2 inhibitors or GLP-1 receptor agonists eTable 10. Lab [file jamanetwopen-e2335797-s001.pdf]

Supplemental Online Content

Ko HY, Bea S, Jeong HE, et al. Sodium-glucose cotransporter 2 inhibitors vs incretin-based drugs and risk of fractures for type 2 diabetes. *JAMA Netw Open*. 2023;6(9):e2335797. doi:10.1001/jamanetworkopen.2023.35797

eFigure 1. Flowchart of study participants selection
eFigure 2. Flowchart of study participants selection
eFigure 3. Cumulative incidence function plots for overall fractures among weighted populations
eFigure 4. Overall study design
eFigure 5. Propensity score distribution of before and after weighting in both cohorts
eTable 1. Baseline characteristics of patients received SGLT2 inhibitors or comparator drugs, before weighting by propensity score fine stratification
eTable 2. Association between the use of SGLT2 inhibitors and the risk of fractures compared with the use of DPP4 inhibitors
eTable 3. Results of subgroup analyses with events, person-years, weighted IRs (per 100 person-years), weighted HRs with 95% CIs, and p for interaction for the association between the use of SGLT2 inhibitors vs. DPP4 inhibitors and the risk of overall fractures
eTable 4. Results of sensitivity analyses with events, person-years, weighted IRs (per 100 person-years), weighted HRs and 95% CIs for the association between the use of SGLT2 inhibitors vs. DPP4 inhibitors and the risk of overall fractures
eTable 5. Association between the use of SGLT2 inhibitors and the risk of fractures compared with the use of GLP-1 receptor agonists
eTable 6. Results of subgroup analyses with events, person-years, weighted IRs (per 100 person-years), weighted HRs with 95% CIs, and p for interaction for the association between the use of SGLT2 inhibitors vs. GLP-1 receptor agonists and the risk of overall fractures
eTable 7. Results of sensitivity analyses with events, person-years, weighted IRs (per 100 person-years), weighted HRs and 95% CIs for the association between the use of SGLT2 inhibitors vs. GLP-1 receptor agonists and the risk of overall fractures
eTable 8. Reasons for the end of follow-up among weighted populations received SGLT2 inhibitors or DPP4 inhibitors
eTable 9. Reasons for the end of follow-up among weighted populations received SGLT2 inhibitors or GLP-1 receptor agonists
eTable 10. Laboratory test results and missing rates of patients receiving SGLT2 inhibitors or comparator drugs before propensity score based fine stratification
eTable 11. Baseline characteristics after weighting by propensity score stratification in patients receiving SGLT2 inhibitors or comparator drugs with bone mineral density test results present
eTable 12. Baseline characteristics before weighting by propensity score stratification in patients receiving SGLT2 inhibitors or comparator drugs with bone mineral density test results present
eTable 13. Association between the use of SGLT2 inhibitors and the risk of overall fractures among weighted populations with bone mineral density test results present
eTable 14. International Classification of Diseases-10 (ICD-10) and domestic procedure codes for identification of fractures
eTable 15. International Classification of Diseases-10 (ICD-10) codes for identification of covariates
eTable 16. Anatomical Therapeutic Chemical (ATC) classification codes for identification of covariates

This supplemental material has been provided by the authors to give readers additional information about their work.

© 2023 Ko HY et al. *JAMA Network Open.*

eTable 1. Baseline characteristics of patients received SGLT2 inhibitors or comparator drugs, before weighting by propensity score fine stratification.

Baseline characteristics	Patients, No. (%)					
	SGLT2i vs. DPP4i			SGLT2i vs. GLP1RA		
	SGLT2i (n=37,532)	DPP4i (n=332,038)	ASD	SGLT2i (n=113,622)	GLP1RA (n=8,181)	ASD
**Cohort entry year**						
2014	1232 (3.3)	18218 (5.5)	0.108	2999 (2.6)	17 (0.2)	0.206
2015	3922 (10.4)	65460 (19.7)	0.261	10701 (9.4)	105 (1.3)	0.368
2016	5220 (13.9)	60320 (18.2)	0.116	16847 (14.8)	703 (8.6)	0.195
2017	6310 (16.8)	52029 (15.7)	0.031	19557 (17.2)	1745 (21.3)	0.105
2018	5815 (15.5)	47041 (14.2)	0.037	18337 (16.1)	1970 (24.1)	0.199
2019	7415 (19.8)	44649 (13.4)	0.17	23214 (20.4)	2076 (25.4)	0.118
2020	7618 (20.3)	44321 (13.3)	0.187	21967 (19.3)	1565 (19.1)	0.005
**Age, mean (SD), y**	60.6 (9.7)	66.0 (11)	0.528	61.4 (9.8)	62.5 (10.2)	0.113
**Age group**			0.460			0.116
45-65	26761 (71.3)	163966 (49.4)		77299 (68.0)	5114 (62.7)	
>65	10771 (28.7)	168072 (50.6)		36323 (32.0)	3067 (37.5)	
**Diabetic medications**						
Alpha-glucosidase inhibitors	1141 (3)	16328 (4.9)	0.096	3151 (2.8)	249 (3)	0.016
GLP1RA	126 (0.3)	262 (0.1)	0.056			
DPP4i				68438 (60.2)	6592 (80.6)	0.457
Insulin	3260 (8.7)	34351 (10.3)	0.057	15720 (13.8)	4050 (49.5)	0.830
Meglitinides	191 (0.5)	2845 (0.9)	0.042	580 (0.5)	113 (1.4)	0.090
Metformin	21189 (56.5)	205528 (61.9)	0.111	90862 (80)	7137 (87.2)	0.197
Sulfonylureas	9388 (25)	112599 (33.9)	0.196	53297 (46.9)	5446 (66.6)	0.405
Thiazolidinediones	2709 (7.2)	17376 (5.2)	0.082	15910 (14)	1502 (18.4)	0.119
**Number of diabetic medications being taken**						
0-1	26470 (70.5)	210488 (63.4)	0.152	31141 (27.4)	481 (5.9)	0.604
2-3	10753 (28.7)	118182 (35.6)	0.149	69321 (61)	5388 (65.9)	0.101
4+	309 (0.8)	3368 (1.0)	0.02	13160 (11.6)	2312 (28.3)	0.427
**Level of diabetes treatment**[a]						
1	25926 (69.1)	205195 (61.8)	0.153	30405 (26.8)	307 (3.8)	0.675
2	8346 (22.2)	92492 (27.9)	0.13	67497 (59.4)	3824 (46.7)	0.256
3	3260 (8.7)	34351 (10.3)	0.057	15720 (13.8)	4050 (49.5)	0.830
**Diabetes related conditions**						
Diabetic nephropathy	1148 (3.1)	11072 (3.3)	0.016	5837 (5.1)	818 (10)	0.185
Diabetic neuropathy	4595 (12.2)	46904 (14.1)	0.056	20766 (18.3)	2300 (28.1)	0.235
Diabetic retinopathy	5730 (15.3)	57925 (17.4)	0.059	25807 (22.7)	3241 (39.6)	0.371
Hypoglycaemia	130 (0.3)	2292 (0.7)	0.048	549 (0.5)	57 (0.7)	0.028
**Charlson Comorbidity Index**						
0	19584 (52.2)	162136 (48.8)	0.067	50365 (44.3)	2438 (29.8)	0.304
1	10586 (28.2)	100633 (30.3)	0.046	41092 (36.2)	4063 (49.7)	0.275
2	4385 (11.7)	38384 (11.6)	0.004	11040 (9.7)	564 (6.9)	0.102
≥3	2977 (7.9)	30885 (9.3)	0.049	11125 (9.8)	1116 (13.6)	0.120
**Number of outpatients visits**						
0-2	1038 (2.8)	11288 (3.4)	0.037	1458 (1.3)	48 (0.6)	0.072
3-5	1738 (4.6)	13108 (3.9)	0.034	3146 (2.8)	96 (1.2)	0.115
6+	34756 (92.6)	307642 (92.7)	0.002	109018 (95.9)	8037 (98.2)	0.137
**Number of hospitalizations**						
0	30119 (80.2)	257820 (77.6)	0.064	86862 (76.4)	5558 (67.9)	0.191
1-2	6815 (18.2)	67448 (20.3)	0.055	24012 (21.1)	2298 (28.1)	0.162
3+	598 (1.6)	6770 (2)	0.033	2748 (2.4)	325 (4)	0.088
**Comorbidities**						
Asthma	3250 (8.7)	30065 (9.1)	0.014	9703 (8.5)	698 (8.5)	<0.001
Chronic kidney disease	201 (0.5)	4336 (1.3)	0.081	1161 (1)	345 (4.2)	0.201
Congestive heart failure	1482 (3.9)	14040 (4.2)	0.014	4847 (4.3)	362 (4.4)	0.008
COPD	2036 (5.4)	21095 (6.4)	0.039	6282 (5.5)	477 (5.8)	0.013
Dementia	1002 (2.7)	20174 (6.1)	0.167	3726 (3.3)	446 (5.5)	0.106
Epilepsy	227 (0.6)	2318 (0.7)	0.012	692 (0.6)	72 (0.9)	0.032
Gout	260 (0.7)	2525 (0.8)	0.008	835 (0.7)	80 (1)	0.026

© 2023 Ko HY et al. *JAMA Network Open.*

Baseline characteristics	Patients, No. (%)					
	SGLT2i vs. DPP4i			SGLT2i vs. GLP1RA		
	SGLT2i (n=37,532)	DPP4i (n=332,038)	ASD	SGLT2i (n=113,622)	GLP1RA (n=8,181)	ASD
Hyperlipidemia	16976 (45.2)	130874 (39.4)	0.118	53250 (46.9)	4065 (49.7)	0.057
Hypertension	20255 (54)	186083 (56)	0.042	61347 (54)	4082 (49.9)	0.082
Ischemic heart disease	3274 (8.7)	27042 (8.1)	0.021	11248 (9.9)	906 (11.1)	0.038
Liver cirrhosis	144 (0.4)	2047 (0.6)	0.033	640 (0.6)	77 (0.9)	0.044
Osteoarthritis	12547 (33.4)	121104 (36.5)	0.064	39159 (34.5)	2939 (35.9)	0.031
Osteoporosis	3268 (8.7)	41189 (12.4)	0.121	10149 (8.9)	766 (9.4)	0.015
Paget's disease	0 (0)	2 (0)	0.003	0 (0)	0 (0)	
Parkinson's disease	129 (0.3)	2748 (0.8)	0.063	502 (0.4)	63 (0.8)	0.042
Rheumatoid arthritis	779 (2.1)	7853 (2.4)	0.02	2405 (2.1)	192 (2.3)	0.016
Stroke	1068 (2.8)	16715 (5)	0.113	3966 (3.5)	391 (4.8)	0.065
Thyroid disease	2951 (7.9)	21697 (6.5)	0.051	8269 (7.3)	622 (7.6)	0.012
**Fall related medications**						
ACE inhibitors or ARBs	19036 (50.7)	161173 (48.5)	0.044	61668 (54.3)	4847 (59.2)	0.100
Anticholinergics	30175 (80.4)	265609 (80)	0.01	92815 (81.7)	6721 (82.2)	0.012
Benzodiazepines	13566 (36.1)	128618 (38.7)	0.054	42268 (37.2)	3150 (38.5)	0.027
beta-blockers	1848 (4.9)	16078 (4.8)	0.004	5599 (4.9)	424 (5.2)	0.012
Calcium channel blockers	14619 (39)	137681 (41.5)	0.051	44490 (39.2)	3262 (39.9)	0.015
Diuretics	9572 (25.5)	88296 (26.6)	0.025	29395 (25.9)	2014 (24.6)	0.029
Nitrates	470 (1.3)	3923 (1.2)	0.006	1664 (1.5)	147 (1.8)	0.026
Opioids	2876 (7.7)	24502 (7.4)	0.011	9761 (8.6)	822 (10)	0.050
Sedative hypnotics	3976 (10.6)	37574 (11.3)	0.023	12690 (11.2)	1067 (13)	0.057
Tricyclic antidepressant	2633 (7)	27112 (8.2)	0.043	9176 (8.1)	891 (10.9)	0.096
Typical antipsychotics	1227 (3.3)	12431 (3.7)	0.026	3913 (3.4)	386 (4.7)	0.064
**Osteoporosis medications**						
Bisphosphonates	1738 (4.6)	25361 (7.6)	0.126	5645 (5)	451 (5.5)	0.024
Calcium/vitamin D	2904 (7.7)	29980 (9)	0.047	9440 (8.3)	892 (10.9)	0.088
Parathyroid hormone/calcitonin	109 (0.3)	1725 (0.5)	0.036	394 (0.3)	21 (0.3)	0.016
RANKL inhibitors	134 (0.4)	1105 (0.3)	0.004	470 (0.4)	43 (0.5)	0.016
SERMs	440 (1.2)	4719 (1.4)	0.022	1357 (1.2)	129 (1.6)	0.033
**Comedications**						
Anticoagulants	1287 (3.4)	11368 (3.4)	<0.001	4680 (4.1)	374 (4.6)	0.022
Anticonvulsants	4302 (11.5)	42083 (12.7)	0.037	17418 (15.3)	2042 (25)	0.242
Antidepressants	2806 (7.5)	26310 (7.9)	0.017	9304 (8.2)	864 (10.6)	0.081
Aromatase inhibitors	2 (0)	5 (0)	0.007	4 (0)	0 (0)	0.008
Oral corticosteroids	14827 (39.5)	125659 (37.8)	0.034	42449 (37.4)	2784 (34)	0.070
Hormone replacement therapy	2180 (5.8)	14532 (4.4)	0.065	6406 (5.6)	479 (5.9)	0.009
Immunosuppressants	114 (0.3)	1670 (0.5)	0.031	384 (0.3)	53 (0.6)	0.044
NSAIDs	25972 (69.2)	228672 (68.9)	0.007	80339 (70.7)	5877 (71.8)	0.025
Platelet inhibitors	23794 (63.4)	222651 (67.1)	0.077	76722 (67.5)	5960 (72.9)	0.117
Proton-pump inhibitors	14687 (39.1)	121518 (36.6)	0.052	47026 (41.4)	3713 (45.4)	0.081
Statins	22389 (59.7)	186734 (56.2)	0.069	80362 (70.7)	6649 (81.3)	0.249

Abbreviations: ACE, angiotensin converting enzyme; ARBs, angiotensin receptor blockers; ASD, absolute standardized difference; COPD, chronic obstructive pulmonary disorder; DPP4i, dipeptidyl peptidase 4 inhibitors; GLP1RA, glucagon like peptide 1 receptor agonists; IQR, interquartile range; NSAIDs, nonsteroidal anti-inflammatory drugs; RANKL, receptor activator of nuclear factors kappa B ligand; SD, standard deviation; SERMs, selective estrogen receptor modulator; SGLT2i, sodium glucose cotransporter 2 inhibitors

[a]Defined depending on the number of antidiabetic medication (excluding the study drugs of interest), prescribed in the year preceding the index date: level 1, as taking none or only one class of antidiabetic medication other than insulin, level 2, as taking ≥2 different classes of antidiabetic medication without insulin, and level 3, as taking insulin with or without other classes of antidiabetic medication.

© 2023 Ko HY et al. *JAMA Network Open.*

eFigure 1. Flowchart of study participants selection

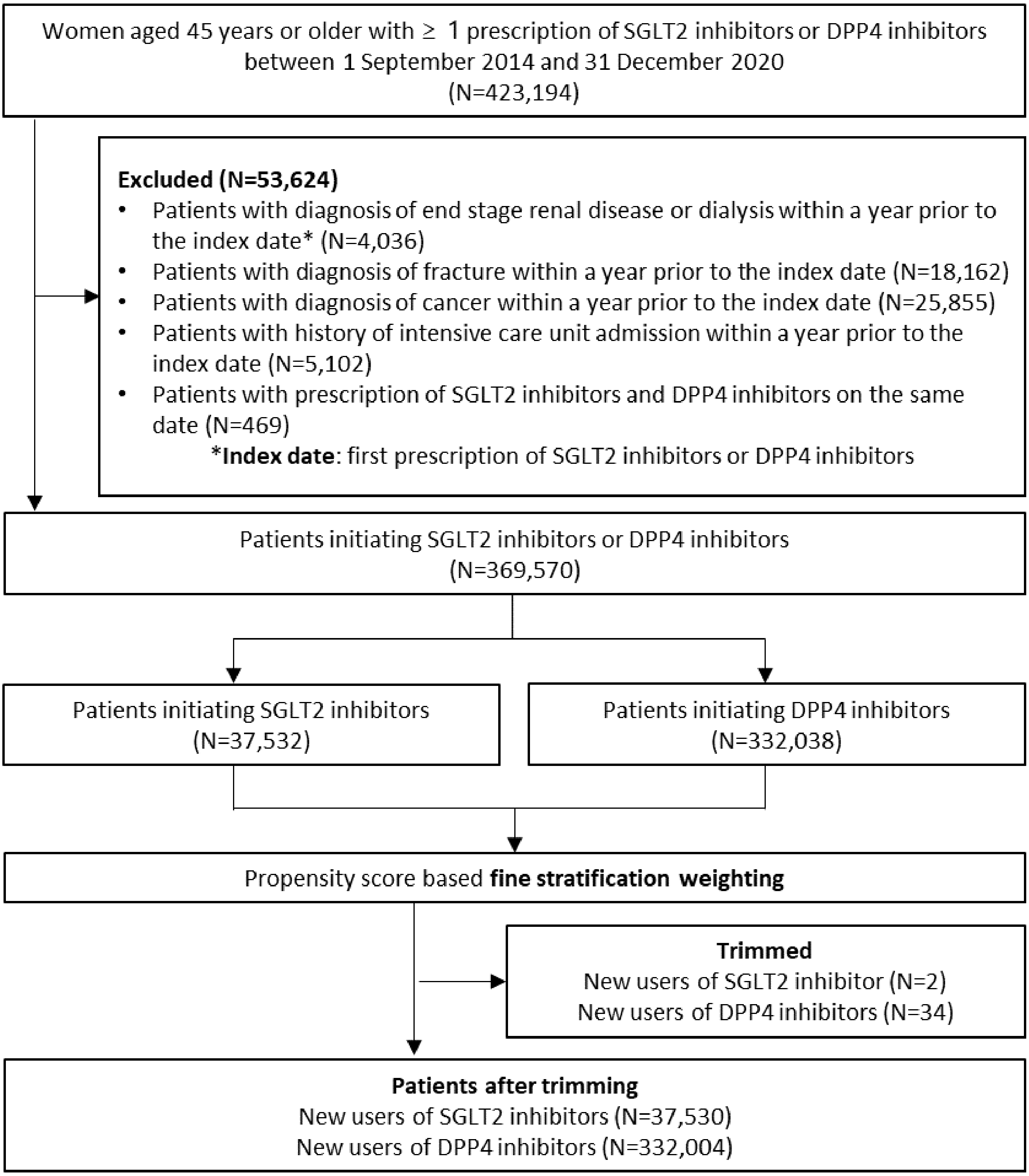

Abbreviations: DPP4, dipeptidyl peptidase 4; SGLT2, sodium glucose cotransporter 2

© 2023 Ko HY et al. *JAMA Network Open.*

eTable 2. Association between the use of SGLT2 inhibitors and the risk of fractures compared with the use of DPP4 inhibitors.

Outcome	SGLT2 inhibitors			DPP4 inhibitors			Weighted HR (95% CI)
	Events	Person-years	Weighted IR[a] (95% CI)	Events	Person-years	Weighted IR[a] (95% CI)	
**Primary outcome**							
Overall fractures	768	54,619	1.41 (1.31-1.51)	18,800	693,818	1.81 (1.78-1.85)	0.78 (0.72-0.84)
**Secondary outcomes**							
Vertebral fractures	376	55,121	0.68 (0.61-0.75)	10,540	707,825	0.96 (0.93-0.98)	0.71 (0.64-0.79)
Hip fractures	82	55,531	0.15 (0.12-0.18)	3,532	720,842	0.25 (0.23-0.26)	0.61 (0.49-0.76)
Humerus fractures	67	55,512	0.12 (0.09-0.15)	1,647	722,725	0.18 (0.16-0.19)	0.70 (0.54-0.89)
Distal radius fractures	262	55,237	0.47 (0.42-0.54)	4,559	716,789	0.52 (0.50-0.53)	0.93 (0.82-1.05)

Abbreviations: CI, confidence interval; DPP4, dipeptidyl peptidase 4; HR, hazard ratio; IR, incidence rate; PS, propensity score; SGLT2, sodium glucose cotransporter 2
[a]per 100 person-years

© 2023 Ko HY et al. *JAMA Network Open.*

eTable 3. Results of subgroup analyses with events, person-years, weighted IRs (per 100 person-years), weighted HRs with 95% CIs, and p for interaction for the association between the use of SGLT2 inhibitors vs. DPP4 inhibitors and the risk of overall fractures.

Stratification	SGLT2 inhibitors			DPP4 inhibitors			Weighted HR (95% CI)	P for interaction
	Events	Person-years	Weighted IR[a] (95% CI)	Events	Person-years	Weighted IR[a] (95% CI)		
**Age group**								
45-60	236	31,585	0.75 (0.65-0.85)	2,178	244,077	0.80 (0.76-0.84)	0.95 (0.83-1.09)	
61-75	363	19,538	1.86 (1.67-2.06)	8,692	315,431	2.49 (2.43-2.55)	0.75 (0.67-0.83)	0.0196
>75	169	3,496	4.84 (4.14-5.63)	7,930	134,309	5.59 (5.46-5.73)	0.85 (0.73-0.99)	0.5097
**Diagnosis of osteoporosis**								
Yes	100	4,128	2.42 (1.97-2.95)	3,622	83,787	3.49 (3.35-3.63)	0.69 (0.56-0.84)	0.3542
No	668	50,492	1.32 (1.22-1.43)	15,178	610,031	1.66 (1.62-1.69)	0.80 (0.74-0.87)	
**Osteoporosis medication use**								
Yes	136	5,998	2.27 (1.90-2.68)	4,463	107,033	3.18 (3.07-3.30)	0.71 (0.60-0.85)	0.3447
No	632	48,621	1.30 (1.20-1.41)	14,337	586,785	1.63 (1.59-1.66)	0.80 (0.74-0.87)	
**Diagnosis of osteoarthritis**								
Yes	315	17,603	1.79 (1.60-2.00)	8,733	250,381	2.54 (2.47-2.60)	0.70 (0.63-0.79)	**0.0203**
No	453	37,016	1.22 (1.11-1.34)	10,067	443,437	1.45 (1.41-1.49)	0.85 (0.77-0.93)	
**Neurological dysfunction**								
**Yes**	91	2,741	3.32 (2.67-4.08)	3,510	63,377	4.29 (4.12-4.45)	0.76 (0.62-0.94)	0.7675
**No**	677	51,879	1.30 (1.21-1.41)	15,290	630,440	1.68 (1.64-1.71)	0.78 (0.72-0.84)	
**Thiazolidinediones use**								
Yes	76	4,548	1.67 (1.32-2.09)	1,423	39,422	2.54 (2.39-2.71)	0.65 (0.52-0.82)	0.1436
No	692	50,071	1.38 (1.28-1.49)	17,377	654,396	1.75 (1.71-1.78)	0.79 (0.73-0.86)	
**Proton-Pump Inhibitor use**								
Yes	316	19,804	1.60 (1.42-1.78)	7,364	236,509	2.17 (2.11-2.24)	0.73 (0.66-0.82)	0.2375
No	452	34,816	1.30 (1.18-1.42)	11,436	457,309	1.61 (1.57-1.65)	0.81 (0.74-0.89)	
**Oral corticosteroids use**								
Yes	314	20,969	1.50 (1.34-1.67)	7,235	256,652	1.93 (1.87-1.98)	0.78 (0.69-0.87)	0.7732
No	454	33,650	1.35 (1.23-1.48)	11,565	437,166	1.74 (1.70-1.78)	0.78 (0.71-0.85)	
**SSRIs use**								
Yes	21	1,153	1.82 (1.13-2.79)	555	13,842	2.51 (2.24-2.82)	0.71 (0.45-1.11)	0.4786
No	747	53,466	1.40 (1.30-1.50)	18,245	679,976	1.80 (1.76-1.83)	0.78 (0.72-0.84)	
**Fall-related medication use**								
Yes	744	51,240	1.45 (1.35-1.56)	18,038	650,753	1.86 (1.82-1.89)	0.78 (0.73-0.84)	0.2449
No	24	3,379	0.71 (0.46-1.06)	762	43,065	1.10 (1.00-1.21)	0.65 (0.43-0.98)	
**Type of SGLT2 inhibitors**								
Dapagliflozin	480	35,021	1.37 (1.25-1.50)	18,800	693,818	1.81 (1.78-1.85)	0.76 (0.69-0.83)	
Empagliflozin	236	15,885	1.49 (1.30-1.69)	18,800	693,818	1.80 (1.76-1.85)	0.82 (0.72-0.94)	
Ipragliflozin	9	494	1.83 (0.83-3.47)	18,800	693,818	1.65 (1.58-1.72)	1.11 (0.58-2.13)	

© 2023 Ko HY et al. *JAMA Network Open.*

Ertugliflozin	43	3,219	1.34 (0.97-1.80)	18,800	693,818	1.81 (1.78-1.84)	0.74 (0.55-0.99)

Abbreviations: CI, confidence interval; DPP4, dipeptidyl peptidase 4; HR, hazard ratio; IR, incidence rate; SGLT2, sodium glucose cotransporter 2; SSRIs, selective serotonin receptor inhibitors

[a]per 100 person-years

© 2023 Ko HY et al. *JAMA Network Open.*

eTable 4. Results of sensitivity analyses with events, person-years, weighted IRs (per 100 person-years), weighted HRs and 95% CIs for the association between the use of SGLT2 inhibitors vs. DPP4 inhibitors and the risk of overall fractures.

Analyses	SGLT2 inhibitors			DPP4 inhibitors			Weighted HR (95% CI)
	Events	Person-years	Weighted IR[a] (95% CI)	Events	Person-years	Weighted IR[a] (95% CI)	
History of surgical menopause	7	717	0.98 (0.39-2.01)	75	5,046	0.79 (0.54-1.13)	1.25 (0.56-2.81)
Hormone replacement therapy	28	3,112	0.90 (0.60-1.30)	384	30,900	1.05 (0.92-1.18)	0.86 (0.58-1.28)
Laboratory results included analysis[b]	768	54,619	1.36 (1.23-1.50)	18,800	693,818	1.74 (1.69-1.78)	0.79 (0.71-0.87)
Extended look-back period[c]	768	54,619	1.41 (1.31-1.51)	18,800	693,818	1.81 (1.78-1.85)	0.78 (0.72-0.84)
Varying grace period							
30-day	649	46,838	1.38 (1.28-1.49)	14,751	565,254	1.73 (1.69-1.77)	0.80 (0.74-0.87)
60-day	731	52,253	1.40 (1.30-1.50)	17,535	654,528	1.78 (1.74-1.81)	0.79 (0.73-0.85)
Stringent outcome definition	289	41,556	0.70 (0.62-0.78)	6,747	522,940	0.86 (0.83-0.88)	0.81 (0.72-0.91)
Control outcomes							
Herpes zoster virus infection	1,563	52,410	2.98 (2.84-3.13)	19,824	687,464	2.89 (2.84-2.93)	1.03 (0.97-1.08)
Hospitalization for heart failure	88	54,287	0.16 (0.13-0.20)	3,121	714,496	0.27 (0.26-0.28)	0.60 (0.48-0.74)

Abbreviations: CI, confidence interval; DPP4, dipeptidyl peptidase 4; HR, hazard ratio; IR, incidence rate; SGLT2, sodium glucose cotransporter 2

[a]per 100 person-years

[b]Laboratory test results were assessed within 3 years prior to the index date and the most recent value was included. Included lab variables comprised waist circumference, body mass index, fasting blood glucose, systolic blood pressure, diastolic blood pressure, total cholesterol, low density lipoprotein cholesterol, high density lipoprotein cholesterol, triglycerides, serum creatinine, estimated glomerular filtration rate, aspartate aminotransferase level, alanine aminotransferase level, gamma glutamyl transferase level, and smoking behavior, which presented varying missing rates of 27.9~57.6% for each variables.

[c]Assessment period for baseline comorbidities was extended to include any time before the index date.

© 2023 Ko HY et al. *JAMA Network Open.*

eFigure 2. Flowchart of study participants selection

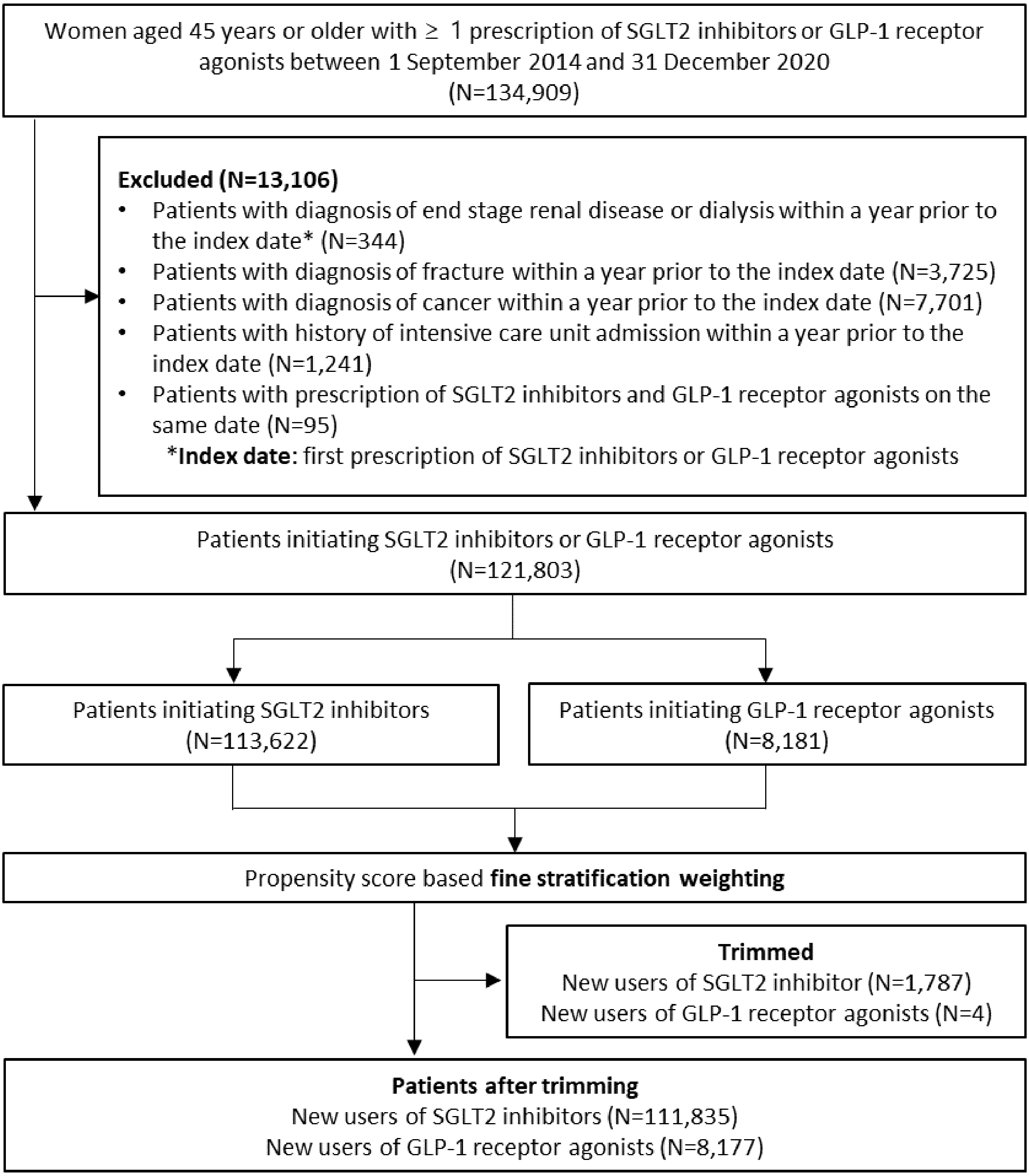

Abbreviations: GLP-1, glucagon like peptide 1; SGLT2, sodium glucose cotransporter 2

© 2023 Ko HY et al. *JAMA Network Open.*

eTable 5. Association between the use of SGLT2 inhibitors and the risk of fractures compared with the use of GLP-1 receptor agonists.

Outcome	SGLT2 inhibitors			GLP-1 receptor agonists			Weighted HR (95% CI)
	Events	Person-years	Weighted IR[a] (95% CI)	Events	Person-years	Weighted IR[a] (95% CI)	
**Primary outcome**							
Overall fractures	2,723	164,967	1.67 (1.60-1.73)	156	6,737	1.92 (1.59-2.30)	0.92 (0.68-1.24)
**Secondary outcomes**							
Vertebral fractures	1,383	166,612	0.84 (0.80-0.88)	66	6,802	0.85 (0.64-1.12)	1.06 (0.67-1.69)
Hip fractures	325	167,928	0.20 (0.18-0.22)	39	6,822	0.38 (0.25-0.57)	0.56 (0.34-0.91)
Humerus fractures	280	167,839	0.17 (0.15-0.19)	26	6,819	0.21 (0.11-0.36)	0.79 (0.43-1.45)
Distal radius fractures	848	167,036	0.51 (0.48-0.55)	34	6,808	0.48 (0.33-0.69)	1.07 (0.54-2.11)

Abbreviations: CI, confidence interval; GLP-1, glucagon like peptide 1; HR, hazard ratio; IR, incidence rate; SGLT2, sodium glucose cotransporter 2
[a]per 100 person-years

© 2023 Ko HY et al. *JAMA Network Open.*

eTable 6. Results of subgroup analyses with events, person-years, weighted IRs (per 100 person-years), weighted HRs with 95% CIs, and p for interaction for the association between the use of SGLT2 inhibitors vs. GLP-1 receptor agonists and the risk of overall fractures.

Stratification	SGLT2 inhibitors			GLP-1 receptor agonists			Weighted HR (95% CI)	P for interaction
	Events	Person-years	Weighted IR[a] (95% CI)	Events	Person-years	Weighted IR[a] (95% CI)		
**Age group**								
45-60	728	92,473	0.79 (0.73-0.85)	21	3,448	0.71 (0.45-1.08)	1.12 (0.59-2.10)	
61-75	1,359	60,789	2.24 (2.12-2.37)	80	2,588	2.67 (2.05-3.42)	0.86 (0.58-1.28)	0.2317
>75	636	11,705	5.39 (4.96-5.86)	55	701	6.42 (4.59-8.74)	0.87 (0.57-1.33)	0.3390
**Diagnosis of osteoporosis**								
Yes	441	12,628	3.62 (3.26-4.01)	25	560	2.39 (1.27-4.08)	1.52 (0.89-2.60)	**0.0064**
No	2,282	152,339	1.52 (1.45-1.58)	131	6,178	1.87 (1.53-2.26)	0.85 (0.63-1.15)	
**Osteoporosis medication use**								
Yes	563	18,924	3.00 (2.75-3.26)	42	939	3.14 (2.05-4.61)	0.98 (0.63-1.52)	0.6910
No	2,160	146,042	1.50 (1.43-1.56)	114	5,798	1.84 (1.49-2.24)	0.85 (0.61-1.20)	
**Diagnosis of osteoarthritis**								
Yes	1,242	54,309	2.31 (2.18-2.45)	64	2,315	1.85 (1.31-2.53)	1.34 (0.93-1.93)	**0.0280**
No	1,481	110,658	1.35 (1.28-1.42)	92	4,423	1.98 (1.57-2.47)	0.71 (0.49-1.04)	
**Neurological dysfunction**								
**Yes**	373	9,432	4.07 (3.65-4.54)	39	594	5.00 (3.32-7.23)	0.84 (0.50-1.39)	0.5315
**No**	2,350	155,535	1.52 (1.46-1.59)	117	6,144	1.62 (1.30-1.99)	0.99 (0.73-1.36)	
**Thiazolidinediones use**								
Yes	459	22,731	2.01 (1.82-2.21)	33	1,331	2.06 (1.37-2.98)	1.06 (0.65-1.73)	0.9328
No	2,264	142,236	1.61 (1.54-1.67)	123	5,407	1.84 (1.48-2.27)	0.91 (0.65-1.28)	
**Proton-Pump Inhibitor use**								
Yes	1,257	62,996	2.01 (1.90-2.13)	85	2,920	2.30 (1.75-2.96)	0.92 (0.66-1.27)	0.9528
No	1,466	101,971	1.46 (1.38-1.53)	71	3,817	1.69 (1.29-2.17)	0.91 (0.51-1.61)	
**Oral corticosteroids use**								
Yes	1,094	61,260	1.81 (1.70-1.92)	61	2,304	2.19 (1.58-2.95)	0.87 (0.57-1.33)	0.8003
No	1,629	103,707	1.59 (1.51-1.67)	95	4,433	1.70 (1.33-2.15)	0.99 (0.68-1.45)	
**SSRIs use**								
Yes	77	3,548	1.98 (1.46-2.62)	5	190	3.74 (1.51-7.71)	0.53 (0.16-1.72)	0.2345
No	2,646	161,419	1.66 (1.59-1.72)	151	6,548	1.96 (1.62-2.35)	0.89 (0.65-1.21)	
**Fall-related medication use**								
Yes	2,652	155,910	1.72 (1.65-1.79)	151	6,443	2.04 (1.69-2.44)	0.89 (0.64-1.23)	0.8225
No	71	9,056	0.81 (0.62-1.05)	5	294	1.47 (0.40-3.77)	0.57 (0.17-1.89)	
**Type of SGLT2 inhibitors**								
Dapagliflozin	1,664	103,612	1.62 (1.55-1.71)	156	6,737	1.85 (1.53-2.23)	0.93 (0.68-1.27)	
Empagliflozin	919	52,185	1.76 (1.65-1.88)	156	6,737	1.98 (1.64-2.37)	0.95 (0.71-1.26)	
Ipragliflozin	15	1,120	1.34 (0.75-2.21)	156	6,737	2.00 (1.51-2.59)	0.76 (0.30-1.91)	

© 2023 Ko HY et al. *JAMA Network Open.*

Ertugliflozin	125	8,050	1.56 (1.30-1.87)	156	6,737	1.76 (1.44-2.12)	0.92 (0.56-1.51)

Abbreviations: CI, confidence interval; GLP-1, glucagon like peptide 1; HR, hazard ratio; IR, incidence rate; SGLT2, sodium glucose cotransporter 2; SSRIs, selective serotonin receptor inhibitors.
[a]per 100 person-years

© 2023 Ko HY et al. *JAMA Network Open.*

eTable 7. Results of sensitivity analyses with events, person-years, weighted IRs (per 100 person-years), weighted HRs and 95% CIs for the association between the use of SGLT2 inhibitors vs. GLP-1 receptor agonists and the risk of overall fractures.

Analyses	SGLT2 inhibitors			GLP-1 receptor agonists			Weighted HR (95% CI)
	Events	Person-years	Weighted IR[a] (95% CI)	Events	Person-years	Weighted IR[a] (95% CI)	
History of surgical menopause	19	2,190	0.61 (0.24-1.26)	0	113	-	-
Hormone replacement therapy	82	9,438	0.89 (0.70-1.12)	4	391	3.74 (1.99-6.39)	0.23 (0.05-1.08)
Laboratory results included analysis[b]	2,723	164,967	1.56 (1.48-1.65)	156	6,737	1.77 (1.30-2.34)	0.92 (0.56-1.51)
Extended look-back period[c]	2,723	164,967	1.66 (1.60-1.72)	156	6,737	1.88 (1.55-2.25)	0.94 (0.69-1.28)
Varying grace period							
30-day	2,194	136,530	1.62 (1.55-1.69)	45	1,809	1.97 (1.38-2.73)	0.95 (0.54-1.66)
60-day	2,529	155,009	1.64 (1.58-1.71)	106	4,777	1.99 (1.60-2.44)	0.88 (0.62-1.27)
Stringent outcome definition	1,004	123,287	0.82 (0.77-0.87)	63	4,800	1.29 (0.97-1.68)	0.69 (0.47-1.01)
Control outcomes							
Herpes zoster virus infection	5,015	158,727	3.18 (3.09-3.27)	220	6,564	3.01 (2.58-3.48)	1.08 (0.86-1.35)
Hospitalization for heart failure	343	164,154	0.21 (0.19-0.24)	27	6,709	0.23 (0.13-0.38)	1.13 (0.66-1.95)

Abbreviations: CI, confidence interval; GLP-1, glucagon like peptide 1; HR, hazard ratio; IR, incidence rate; SGLT2, sodium glucose cotransporter 2; SSRIs, selective serotonin receptor inhibitors

[a]per 100 person-years

[b]Laboratory test results were assessed within 3 years prior to the index date and the most recent value was included. Included lab variables comprised waist circumference, body mass index, fasting blood glucose, systolic blood pressure, diastolic blood pressure, total cholesterol, low density lipoprotein cholesterol, high density lipoprotein cholesterol, triglycerides, serum creatinine, estimated glomerular filtration rate, aspartate aminotransferase level, alanine aminotransferase level, gamma glutamyl transferase level, and smoking behavior, which presented varying missing rates of 27.9~60.5% for each variables.

[c]Assessment period for baseline comorbidities was extended to include any time before the index date.

© 2023 Ko HY et al. *JAMA Network Open.*

eTable 8. Reasons for the end of follow-up among weighted populations received SGLT2 inhibitors or DPP4 inhibitors.

Reasons	SGLT2 inhibitors (n=37,530)	DPP4 inhibitors (n=332,004)
Follow-up, median (IQR), years	0.91 (0.35-2.15)	1.52 (0.49-3.42)
Follow-up, mean (SD), years	1.45 (1.42)	2.09 (1.79)
Outcome occurrence, n (%)	768 (2.05 %)	18,794 (5.66%)
Drug discontinuation, n (%)	9,610 (25.61 %)	110,836 (33.38%)
Drug switch/add-on to comparator drug, n (%)	9,385 (25.01 %)	24,079 (7.25%)
Death, n (%)	171 (0.46 %)	8,317 (2.51%)
End of the study period, n (%)	17,596 (46.89 %)	169,978 (51.20%)

Abbreviations: DPP4, dipeptidyl peptidase 4; IQR, interquartile range; SD, standard deviation; SGLT2, sodium glucose cotransporter 2

© 2023 Ko HY et al. *JAMA Network Open.*

eTable 9. Reasons for the end of follow-up among weighted populations received SGLT2 inhibitors or GLP-1 receptor agonists.

Reasons	SGLT2 inhibitors (n=111,835)	GLP-1 receptor agonists (n=8,177)
Follow-up, median (IQR), years	0.92 (0.41-2.06)	0.48 (0.25-1.05)
Follow-up, mean (SD), years	1.43 (1.33)	0.82 (0.85)
Outcome occurrence, n (%)	2,673 (2.39 %)	156 (1.91 %)
Drug discontinuation, n (%)	55,126 (49.29 %)	5,373 (65.71 %)
Drug switch/add-on to comparator drug, n (%)	2,165 (1.94 %)	576 (7.04 %)
Death, n (%)	665 (0.59 %)	48 (0.59 %)
End of the study period, n (%)	51,206 (45.79 %)	2,024 (24.75 %)

Abbreviations: GLP-1, glucagon like peptide 1; IQR, interquartile range; SD, standard deviation; SGLT2, sodium glucose cotransporter 2

© 2023 Ko HY et al. *JAMA Network Open.*

eTable 10. Laboratory test results and missing rates of patients receiving SGLT2 inhibitors or comparator drugs before propensity score based fine stratification.

Baseline characteristics[a]	SGLT2i vs. DPP4i			SGLT2i vs. GLP1RA		
	**SGLT2i (n=37,532)**	**DPP4i (n=332,038)**	**ASD**	**SGLT2i (n=113,622)**	**GLP1RA (n=8,181)**	**ASD**
**Laboratory test results, mean (SD)**						
Waist circumference [cm]	87.2 (9.5)	84.4 (9.6)	0.293	86.5 (9.6)	86.7 (10.4)	0.021
Missing; n (%)	10480 (27.9)	113017 (34)	0.133	32025 (28.2)	2541 (31.1)	0.063
Body mass index [kg/m^2]	27.4 (4.1)	25.7 (6.3)	0.321	26.8 (4)	26.5 (4.5)	0.087
Missing; n (%)	10481 (27.9)	113058 (34)	0.133	32027 (28.2)	2541 (31.1)	0.063
Fasting blood glucose [mg/dL]	142.4 (47.6)	143.6 (49.4)	0.024	151.3 (52.4)	163.1 (61.8)	0.207
Missing; n (%)	10616 (28.3)	114626 (34.5)	0.135	32642 (28.7)	2620 (32)	0.072
Systolic blood pressure [mmHg]	129.4 (15.5)	129.4 (15.7)	0.002	128.5 (15.4)	127.5 (16)	0.063
Missing; n (%)	10615 (28.3)	114599 (34.5)	0.135	32634 (28.7)	2617 (32)	0.071
Diastolic blood pressure [mmHg]	78.3 (10)	77.5 (9.9)	0.086	77.2 (9.8)	75.4 (10)	0.185
Missing; n (%)	10615 (28.3)	114599 (34.5)	0.135	32634 (28.7)	2617 (32)	0.071
Total cholesterol. [mg/dL]	199.8 (52.7)	197.3 (49)	0.049	187.3 (50.3)	174.3 (44.8)	0.273
Missing; n (%)	16808 (44.8)	149130 (44.9)	0.003	50282 (44.3)	3874 (47.4)	0.062
Low density lipoprotein cholesterol [mg/dL]	114.4 (49.2)	112.9 (44.1)	0.032	103.7 (45.5)	92.8 (38.9)	0.258
Missing; n (%)	17059 (45.5)	150512 (45.3)	0.002	50897 (44.8)	3916 (47.9)	0.062
High density lipoprotein cholesterol [mg/dL]	53.7 (14.5)	53.5 (16.9)	0.012	52.6 (13.7)	51.3 (12.8)	0.102
Missing; n (%)	16808 (44.8)	149130 (44.9)	0.003	50282 (44.3)	3874 (47.4)	0.062
Triglycerides [mg/dL]	164.5 (112.5)	158.4 (106.4)	0.055	159.6 (110.6)	157.1 (127.2)	0.02
Missing; n (%)	16808 (44.8)	149130 (44.9)	0.003	50282 (44.3)	3874 (47.4)	0.062
Serum creatinine [mg/dL]	0.8 (0.6)	0.8 (0.5)	0.051	0.8 (0.6)	0.8 (0.3)	0.099
Missing; n (%)	10615 (28.3)	114630 (34.5)	0.135	32641 (28.7)	2620 (32)	0.072
eGFR [mL/min/1.73m^2]	88.4 (25.7)	84.6 (26.7)	0.148	87.8 (26.4)	84.8 (31.1)	0.102
Missing; n (%)	21629 (57.6)	176777 (53.2)	0.088	64070 (56.4)	4953 (60.5)	0.084
AST (SGOT) [IU/L]	32.2 (24.8)	29.7 (25.4)	0.098	31.3 (24.1)	29.9 (21.4)	0.06
Missing; n (%)	10615 (28.3)	114628 (34.5)	0.135	32641 (28.7)	2620 (32)	0.072
ALT (SGPT) [IU/L]	35.2 (29.6)	30.5 (28.9)	0.16	33.3 (28.8)	30.3 (23.7)	0.111
Missing; n (%)	10615 (28.3)	114633 (34.5)	0.135	32642 (28.7)	2620 (32)	0.072
GGT [IU/L]	41.8 (44.3)	37.3 (46.8)	0.097	39.5 (44.3)	36.8 (40.7)	0.065
Missing; n (%)	10615 (28.3)	114628 (34.5)	0.135	32641 (28.7)	2620 (32)	0.072
**Lifestyle risk factors, n (%)**						
Smoking	771 (2.8)	5814 (2.7)	0.012	2344 (2.9)	179 (3.2)	0.018
Missing; n (%)	10477 (27.9)	112961 (34)	0.132	32014 (28.2)	2539 (31)	0.063

Abbreviations: ASD, absolute standardized difference; AST, aspartate aminotransferase; ALT, alanine aminotransferase; DPP4i, dipeptidyl peptidase 4 inhibitors; eGFR, estimated glomerular filtration rate; GGT, gamma glutamyl transferase; GLP1RA, glucagon like peptide 1 receptor agonists; SD, standard deviation; SGLT2i, sodium glucose cotransporter 2 inhibitors; SGOT, serum glutamic oxaloacetic transaminase; SGPT, serum glutamic pyruvate transaminase.

[a]Baseline characteristics were assessed within 3 years prior to the index date and the most recent value was included.

© 2023 Ko HY et al. *JAMA Network Open.*

eTable 11. Baseline characteristics after weighting by propensity score stratification in patients receiving SGLT2 inhibitors or comparator drugs with bone mineral density test results present.

Baseline characteristics	Patients, No. (%)					
	SGLT2i vs. DPP4i			SGLT2i vs. GLP1RA		
	SGLT2i (n=1,257)	DPP4i (n=6,103)	ASD	SGLT2i (n=2,514)	GLP1RA (n=250)	ASD
**Cohort entry year**						
2018	160 (12.7)	771 (12.6)	0.003	332 (13.2)	27 (10.7)	0.078
2019	496 (39.5)	2416 (39.6)	0.003	1031 (41)	98 (39.4)	0.033
2020	601 (47.8)	2917 (47.8)	<0.001	1151 (45.8)	125 (49.9)	0.083
**Age, mean (SD), y**	60.1 (6)	60.1 (6)	<0.001	61 (6.1)	61.1 (6)	0.012
**Age group**			0.001			0.008
45-65	682 (54.3)	3315 (54.3)		1210 (48.1)	121 (48.5)	
>65	575 (45.7)	2788 (45.7)		1304 (51.9)	129 (51.5)	
**Diabetic medications**						
Alpha-glucosidase inhibitors	14 (1.1)	65 (1.1)	0.004	36 (1.4)	13 (5)	0.204
GLP1 RAs	2 (0.2)	9 (0.1)	0.004			
DPP4 inhibitors				1786 (71)	179 (71.4)	0.009
Insulin	48 (3.8)	234 (3.8)	0.001	340 (13.5)	37 (15)	0.042
Meglitinides	2 (0.2)	9 (0.1)	0.005	7 (0.3)	0 (0.1)	0.046
Metformin	632 (50.3)	3061 (50.2)	0.002	2148 (85.4)	220 (87.9)	0.074
Sulfonylureas	178 (14.2)	860 (14.1)	0.002	1226 (48.8)	124 (49.6)	0.016
Thiazolidinediones	56 (4.5)	276 (4.5)	0.003	380 (15.1)	48 (19.3)	0.11
**Number of diabetic medications being taken**						
0-1	1044 (83.1)	5075 (83.1)	0.003	466 (18.5)	45 (18.1)	0.011
2-3	210 (16.7)	1014 (16.6)	0.002	1791 (71.2)	166 (66.5)	0.102
4+	3 (0.2)	14 (0.2)	0.001	257 (10.2)	38 (15.4)	0.155
**Level of diabetes treatment**						
1	1032 (82.1)	5013 (82.1)	0.001	453 (18)	44 (17.6)	0.01
2	177 (14.1)	855 (14)	0.002	1721 (68.5)	168 (67.4)	0.023
3	48 (3.8)	234 (3.8)	0.001	340 (13.5)	37 (15)	0.042
**Diabetes related conditions**						
Diabetic nephropathy	29 (2.3)	145 (2.4)	0.005	133 (5.3)	11 (4.3)	0.045
Diabetic neuropathy	117 (9.3)	582 (9.5)	0.008	476 (18.9)	49 (19.4)	0.012
Diabetic retinopathy	162 (12.9)	793 (13)	0.003	666 (26.5)	58 (23.1)	0.08
Hypoglycaemia	3 (0.2)	12 (0.2)	0.009	6 (0.2)	0 (0.1)	0.039
**Charlson Comorbidity Index**						
0	680 (54.1)	3286 (53.8)	0.005	1136 (45.2)	92 (36.9)	0.17
1	306 (24.3)	1485 (24.3)	<0.001	899 (35.8)	105 (42)	0.128
2	170 (13.5)	834 (13.7)	0.004	256 (10.2)	32 (12.7)	0.08
≥3	101 (8)	498 (8.2)	0.005	223 (8.9)	21 (8.4)	0.016
**Number of outpatients visits**						
0-2	22 (1.8)	107 (1.8)	<0.001	0 (0)	0 (0)	
3-5	57 (4.5)	285 (4.7)	0.007	57 (2.3)	3 (1.2)	0.085
6+	1178 (93.7)	5711 (93.6)	0.006	2457 (97.7)	247 (98.8)	0.085
**Number of hospitalizations**						
0	1011 (80.4)	4907 (80.4)	0.001	1919 (76.3)	175 (70.1)	0.142
1-2	223 (17.7)	1080 (17.7)	0.001	527 (21)	63 (25.2)	0.101
3+	23 (1.8)	116 (1.9)	0.005	68 (2.7)	12 (4.7)	0.106
**Comorbidities**						
Asthma	107 (8.5)	528 (8.6)	0.005	186 (7.4)	22 (8.8)	0.05
Chronic kidney disease	14 (1.1)	73 (1.2)	0.007	26 (1)	1 (0.3)	0.096
Congestive heart failure	30 (2.4)	162 (2.7)	0.017	57 (2.3)	5 (1.8)	0.03
COPD	90 (7.2)	419 (6.9)	0.012	135 (5.4)	17 (6.8)	0.059
Dementia	19 (1.5)	88 (1.4)	0.006	41 (1.6)	8 (3.3)	0.105

© 2023 Ko HY et al. *JAMA Network Open.*

Epilepsy	3 (0.2)	15 (0.2)	<0.001	11 (0.4)	1 (0.2)	0.033
Gout	13 (1)	62 (1)	0.001	23 (0.9)	1 (0.5)	0.05
Hyperlipidemia	651 (51.8)	3157 (51.7)	0.001	1357 (54)	126 (50.4)	0.071

	**Patients, No. (%)**					
	**SGLT2i vs. DPP4i**			**SGLT2i vs. GLP1RA**		
**Baseline characteristics**	**SGLT2i (n=1,257)**	**DPP4i (n=6,103)**	**ASD**	**SGLT2i (n=2,514)**	**GLP1RA (n=250)**	**ASD**
Hypertension	657 (52.3)	3169 (51.9)	0.007	1298 (51.6)	111 (44.5)	0.143
Ischemic heart disease	87 (6.9)	427 (7)	0.003	197 (7.8)	20 (8)	0.008
Liver cirrhosis	4 (0.3)	18 (0.3)	0.005	20 (0.8)	4 (1.5)	0.066
Osteoarthritis	477 (37.9)	2313 (37.9)	0.001	960 (38.2)	102 (40.9)	0.055
Osteoporosis	161 (12.8)	775 (12.7)	0.003	325 (12.9)	43 (17.2)	0.12
Parkinson's disease	4 (0.3)	19 (0.3)	0.001	0 (0)	0 (0)	
Rheumatoid arthritis	23 (1.8)	108 (1.8)	0.005	53 (2.1)	4 (1.8)	0.024
Stroke	21 (1.7)	97 (1.6)	0.006	60 (2.4)	5 (2.1)	0.023
Thyroid disease	81 (6.4)	394 (6.5)	0.001	188 (7.5)	24 (9.5)	0.074
**Fall related medications**						
ACE inhibitors or ARBs	603 (48)	2917 (47.8)	0.004	1315 (52.3)	120 (48.1)	0.085
Anticholinergics	1036 (82.4)	5035 (82.5)	0.002	2105 (83.7)	223 (89.3)	0.164
Benzodiazepines	478 (38)	2341 (38.4)	0.007	1029 (40.9)	112 (44.7)	0.076
beta-blockers	54 (4.3)	262 (4.3)	<0.001	118 (4.7)	14 (5.4)	0.033
Calcium channel blockers	438 (34.8)	2097 (34.4)	0.01	888 (35.3)	80 (32.2)	0.066
Diuretics	271 (21.6)	1302 (21.3)	0.005	494 (19.6)	44 (17.4)	0.057
Nitrates	10 (0.8)	51 (0.8)	0.004	32 (1.3)	2 (0.7)	0.055
Opioids	117 (9.3)	568 (9.3)	<0.001	300 (11.9)	39 (15.5)	0.103
Sedative hypnotics	136 (10.8)	679 (11.1)	0.01	277 (11)	25 (9.9)	0.038
Tricyclic antidepressant	77 (6.1)	375 (6.1)	0.001	181 (7.2)	30 (12)	0.164
Typical antipsychotics	36 (2.9)	175 (2.9)	<0.001	78 (3.1)	16 (6.5)	0.161
**Osteoporosis medications**						
Bisphosphonates	69 (5.5)	332 (5.4)	0.002	165 (6.6)	18 (7.1)	0.021
Calcium/vitamin D	132 (10.5)	661 (10.8)	0.011	322 (12.8)	26 (10.4)	0.075
Parathyroid hormone/calcitonin	4 (0.3)	21 (0.3)	0.005	28 (1.1)	9 (3.6)	0.166
RANKL inhibitors	12 (1)	55 (0.9)	0.006			
SERMs	30 (2.4)	147 (2.4)	0.001	59 (2.3)	2 (0.9)	0.118
**Comedications**						
Anticoagulants	37 (2.9)	177 (2.9)	0.003	86 (3.4)	8 (3.1)	0.018
Anticonvulsants	168 (13.4)	822 (13.5)	0.003	455 (18.1)	48 (19.2)	0.029
Antidepressants	111 (8.8)	545 (8.9)	0.004	188 (7.5)	27 (10.8)	0.117
Aromatase inhibitors						
Oral corticosteroids	541 (43)	2631 (43.1)	0.001	958 (38.1)	128 (51.4)	0.27
Hormone replacement therapy	98 (7.8)	471 (7.7)	0.003	190 (7.6)	20 (7.8)	0.011
Immunosuppressants	5 (0.4)	23 (0.4)	0.005	12 (0.5)	0 (0.1)	0.06
NSAIDs	913 (72.6)	4431 (72.6)	0.001	1855 (73.8)	186 (74.4)	0.014
Platelet inhibitors	672 (53.5)	3272 (53.6)	0.003	1548 (61.6)	151 (60.5)	0.021
Proton-pump inhibitors	622 (49.5)	3019 (49.5)	<0.001	1346 (53.5)	154 (61.4)	0.16
Statins	760 (60.5)	3677 (60.2)	0.004	1954 (77.7)	186 (74.4)	0.078
**Laboratory test results[a], mean (SD)**						
Waist circumference [cm]	87.2 (9.5)	87.2 (10)	0.002	86 (9.6)	86.5 (9.7)	0.052
Body mass index [kg/m^2]	27.4 (3.9)	27.4 (4.3)	0.003	26.5 (3.9)	27 (4.4)	0.112
Fasting blood glusoce [mg/dL]	145 (46.6)	144.8 (45.7)	0.004	153.3 (51)	151.8 (55.6)	0.028
Systolic blood pressure [mmHg]	130.1 (15.9)	130.1 (16)	0.003	128.5 (15)	128.6 (16)	0.01
Diastolic blood pressure [mmHg]	78.8 (10)	78.8 (10.1)	0.003	76.5 (9.7)	76.5 (9.5)	0.003
Serum creatinine [mg/dL]	0.7 (0.2)	0.7 (0.2)	0.008	0.7 (0.2)	0.7 (0.2)	0.004
AST (SGOT) [IU/L]	34.7 (25.2)	34.6 (23.6)	0.005	31.3 (19.2)	30.9 (17.9)	0.022
ALT (SGPT) [IU/L]	39.3 (36.5)	39 (31.6)	0.008	32.5 (23.3)	31.9 (22.9)	0.024
GGT [IU/L]	45 (42.4)	44.9 (48.5)	0.004	39.6 (42)	40 (58.8)	0.007
**Bone mineral density test results[b]**						
T-score >= -1.0	556 (44.2)	2732 (44.8)	0.011	1032 (41.1)	97 (38.8)	0.047
-2.5 < T-score < -1.0	500 (39.8)	2408 (39.5)	0.006	1025 (40.8)	105 (42)	0.025
T-score <= -2.5	201 (16)	963 (15.8)	0.006	457 (18.2)	48 (19.2)	0.027

© 2023 Ko HY et al. *JAMA Network Open.*

Abbreviations: ASD, absolute standardized difference; AST, aspartate aminotransferase; ALT, alanine aminotransferase; DPP4i, dipeptidyl peptidase 4 inhibitors; GGT, gamma glutamyl transferase; GLP1RA, glucagon like peptide 1 receptor agonists; SD, standard deviation; SGLT2i, sodium glucose cotransporter 2 inhibitors; SGOT, serum glutamic oxaloacetic transaminase; SGPT, serum glutamic pyruvate transaminase.
[a]Laboratory test results were assessed within 3 years prior to the index date and the most recent value was included.
[b]Bone mineral density tests were conducted using DXA (dual-energy X-ray absorptiometry). The test results were assessed within 3 years prior to the index date and the most recent value was included.

© 2023 Ko HY et al. *JAMA Network Open.*

eTable 12. Baseline characteristics before weighting by propensity score stratification in patients receiving SGLT2 inhibitors or comparator drugs with bone mineral density test results present.

	Patients, No. (%)					
	SGLT2i vs. DPP4i			SGLT2i vs. GLP1RA		
**Baseline characteristics**	**SGLT2i (n=1,273)**	**DPP4i (n=6,331)**	**ASD**	**SGLT2i (n=3,574)**	**GLP-1 RA (n=269)**	**ASD**
**Cohort entry year**						
2018	164 (12.9)	1018 (16.1)	0.091	441 (12.3)	44 (16.4)	0.115
2019	499 (39.2)	2308 (36.5)	0.057	1436 (40.2)	119 (44.2)	0.082
2020	610 (47.9)	3005 (47.5)	0.009	1697 (47.5)	106 (39.4)	0.163
**Age, mean (SD), y**	60.2 (6)	61.7 (6)	0.246	60.9 (6.1)	61.6 (6)	0.11
**Age group**			0.238			0.117
45-65	683 (53.7)	2649 (41.8)		1746 (48.6)	115 (42.8)	
>65	590 (46.3)	3682 (58.2)		1838 (51.4)	154 (57.2)	
**Diabetic medications**						
Alpha-glucosidase inhibitors	14 (1.1)	87 (1.4)	0.025	43 (1.2)	5 (1.9)	0.053
GLP1 RAs	2 (0.2)	11 (0.2)	0.004			
DPP4 inhibitors				2052 (57.4)	220 (81.8)	0.549
Insulin	49 (3.8)	249 (3.9)	0.004	367 (10.3)	125 (46.5)	0.877
Meglitinides	2 (0.2)	14 (0.2)	0.015	7 (0.2)	1 (0.4)	0.033
Metformin	638 (50.1)	3470 (54.8)	0.094	2759 (77.2)	238 (88.5)	0.303
Sulfonylureas	180 (14.1)	969 (15.3)	0.033	1336 (37.4)	169 (62.8)	0.526
Thiazolidinediones	57 (4.5)	186 (2.9)	0.082	426 (11.9)	51 (19)	0.196
**Number of diabetic medications being taken**						
0-1	1057 (83)	5233 (82.7)	0.01	1230 (34.4)	16 (5.9)	0.759
2-3	213 (16.7)	1082 (17.1)	0.01	2058 (57.6)	189 (70.3)	0.266
4+	3 (0.2)	16 (0.3)	0.003	286 (8)	64 (23.8)	0.442
**Level of diabetes treatment**						
1	1045 (82.1)	5187 (81.9)	0.004	1216 (34)	11 (4.1)	0.824
2	179 (14.1)	895 (14.1)	0.002	1991 (55.7)	133 (49.4)	0.126
3	49 (3.8)	249 (3.9)	0.004	367 (10.3)	125 (46.5)	0.877
**Diabetes related conditions**						
Diabetic nephropathy	29 (2.3)	137 (2.2)	0.008	156 (4.4)	20 (7.4)	0.131
Diabetic neuropathy	121 (9.5)	671 (10.6)	0.036	593 (16.6)	77 (28.6)	0.291
Diabetic retinopathy	165 (13)	935 (14.8)	0.052	786 (22)	121 (45)	0.502
Hypoglycaemia	3 (0.2)	10 (0.2)	0.018	7 (0.2)	1 (0.4)	0.033
**Charlson Comorbidity Index**						
0	686 (53.9)	3585 (56.6)	0.055	1688 (47.2)	91 (33.8)	0.276
1	309 (24.3)	1545 (24.4)	0.003	1169 (32.7)	119 (44.2)	0.239
2	173 (13.6)	775 (12.2)	0.04	403 (11.3)	25 (9.3)	0.065
≥3	105 (8.2)	426 (6.7)	0.058	314 (8.8)	34 (12.6)	0.125
**Number of outpatients visits**						
0-2	22 (1.7)	134 (2.1)	0.028	29 (0.8)	0 (0)	0.128
3-5	57 (4.5)	248 (3.9)	0.028	95 (2.7)	4 (1.5)	0.082
6+	1194 (93.8)	5949 (94)	0.007	3450 (96.5)	265 (98.5)	0.128
**Number of hospitalizations**						
0	1023 (80.4)	5088 (80.4)	<0.001	2763 (77.3)	182 (67.7)	0.217
1-2	227 (17.8)	1127 (17.8)	0.001	717 (20.1)	76 (28.3)	0.192
3+	23 (1.8)	116 (1.8)	0.002	94 (2.6)	11 (4.1)	0.081
**Comorbidities**						
Asthma	109 (8.6)	453 (7.2)	0.052	288 (8.1)	15 (5.6)	0.099
Chronic kidney disease	14 (1.1)	31 (0.5)	0.069	43 (1.2)	5 (1.9)	0.053
Congestive heart failure	32 (2.5)	135 (2.1)	0.025	98 (2.7)	8 (3)	0.014
COPD	91 (7.1)	354 (5.6)	0.064	235 (6.6)	11 (4.1)	0.111
Dementia	20 (1.6)	141 (2.2)	0.048	58 (1.6)	10 (3.7)	0.13

Epilepsy	4 (0.3)	29 (0.5)	0.023	14 (0.4)	2 (0.7)	0.047
Gout	13 (1)	70 (1.1)	0.008	38 (1.1)	3 (1.1)	0.005
Hyperlipidemia	659 (51.8)	3145 (49.7)	0.042	1888 (52.8)	142 (52.8)	0.001

	**Patients, No. (%)**					
	**SGLT2i vs. DPP4i**			**SGLT2i vs. GLP1RA**		
**Baseline characteristics**	**SGLT2i (n=1,273)**	**DPP4i (n=6,331)**	**ASD**	**SGLT2i (n=3,574)**	**GLP-1 RA (n=269)**	**ASD**
Hypertension	669 (52.6)	3189 (50.4)	0.044	1863 (52.1)	134 (49.8)	0.046
Ischemic heart disease	94 (7.4)	320 (5.1)	0.097	291 (8.1)	30 (11.2)	0.102
Liver cirrhosis	5 (0.4)	37 (0.6)	0.027	28 (0.8)	5 (1.9)	0.094
Osteoarthritis	487 (38.3)	2305 (36.4)	0.038	1395 (39)	99 (36.8)	0.046
Osteoporosis	166 (13)	965 (15.2)	0.063	459 (12.8)	43 (16)	0.09
Parkinson's disease	4 (0.3)	17 (0.3)	0.008	12 (0.3)	1 (0.4)	0.006
Rheumatoid arthritis	23 (1.8)	153 (2.4)	0.042	79 (2.2)	6 (2.2)	0.001
Stroke	22 (1.7)	154 (2.4)	0.049	82 (2.3)	13 (4.8)	0.137
Thyroid disease	84 (6.6)	502 (7.9)	0.051	266 (7.4)	23 (8.6)	0.041
**Fall related medications**						
ACE inhibitors or ARBs	610 (47.9)	2722 (43)	0.099	1844 (51.6)	151 (56.1)	0.091
Anticholinergics	1050 (82.5)	5146 (81.3)	0.031	2964 (82.9)	235 (87.4)	0.125
Benzodiazepines	490 (38.5)	2415 (38.1)	0.007	1422 (39.8)	126 (46.8)	0.143
beta-blockers	56 (4.4)	302 (4.8)	0.018	172 (4.8)	10 (3.7)	0.054
Calcium channel blockers	445 (35)	2199 (34.7)	0.005	1257 (35.2)	112 (41.6)	0.133
Diuretics	276 (21.7)	1235 (19.5)	0.054	763 (21.3)	47 (17.5)	0.098
Nitrates	10 (0.8)	29 (0.5)	0.042	37 (1)	7 (2.6)	0.117
Opioids	120 (9.4)	639 (10.1)	0.022	404 (11.3)	41 (15.2)	0.116
Sedative hypnotics	139 (10.9)	685 (10.8)	0.003	382 (10.7)	39 (14.5)	0.115
Tricyclic antidepressant	80 (6.3)	388 (6.1)	0.006	251 (7)	25 (9.3)	0.083
Typical antipsychotics	36 (2.8)	222 (3.5)	0.039	104 (2.9)	13 (4.8)	0.1
**Osteoporosis medications**						
Bisphosphonates	72 (5.7)	516 (8.2)	0.099	212 (5.9)	22 (8.2)	0.088
Calcium/vitamin D	135 (10.6)	746 (11.8)	0.037	436 (12.2)	48 (17.8)	0.158
Parathyroid hormone/calcitonin	4 (0.3)	28 (0.4)	0.021	12 (0.3)	0 (0)	0.082
RANKL inhibitors	12 (0.9)	78 (1.2)	0.028	42 (1.2)	4 (1.5)	0.027
SERMs	30 (2.4)	139 (2.2)	0.011	74 (2.1)	9 (3.3)	0.079
**Comedications**						
Anticoagulants	41 (3.2)	138 (2.2)	0.064	133 (3.7)	11 (4.1)	0.019
Anticonvulsants	172 (13.5)	770 (12.2)	0.04	580 (16.2)	82 (30.5)	0.342
Antidepressants	113 (8.9)	440 (6.9)	0.071	287 (8)	25 (9.3)	0.045
Oral corticosteroids	548 (43)	2763 (43.6)	0.012	1437 (40.2)	99 (36.8)	0.07
Hormone replacement therapy	98 (7.7)	475 (7.5)	0.007	257 (7.2)	24 (8.9)	0.064
Immunosuppressants	5 (0.4)	40 (0.6)	0.033	17 (0.5)	3 (1.1)	0.072
NSAIDs	925 (72.7)	4476 (70.7)	0.044	2631 (73.6)	204 (75.8)	0.051
Platelet inhibitors	684 (53.7)	3421 (54)	0.006	2106 (58.9)	190 (70.6)	0.247
Proton-pump inhibitors	635 (49.9)	3094 (48.9)	0.02	1870 (52.3)	159 (59.1)	0.137
Statins	773 (60.7)	3699 (58.4)	0.047	2623 (73.4)	228 (84.8)	0.282
**Laboratory test results[a], mean (SD)**						
Waist circumference [cm]	87.2 (9.5)	84.1 (9.2)	0.337	86.3 (9.7)	86.2 (9.9)	0.009
Body mass index [kg/m^2]	27.4 (3.9)	25.8 (3.7)	0.419	26.8 (4)	26.1 (4.1)	0.152
Fasting blood glusoce [mg/dL]	145 (46.6)	146.1 (49.9)	0.023	149.7 (49.1)	161.7 (64.7)	0.209
Systolic blood pressure [mmHg]	130.1 (16)	129.2 (15.8)	0.058	129 (15.3)	126.6 (16.1)	0.151
Diastolic blood pressure [mmHg]	78.7 (10)	77.6 (9.9)	0.118	77.1 (9.8)	74.3 (9.6)	0.29
Serum creatinine [mg/dL]	0.7 (0.2)	0.7 (0.2)	0.034	0.7 (0.2)	0.8 (0.3)	0.209
AST (SGOT) [IU/L]	34.7 (25.1)	32.5 (25.9)	0.087	32.8 (22.6)	30 (18.1)	0.137
ALT (SGPT) [IU/L]	40.1 (47.9)	34.3 (27.4)	0.147	35.2 (34.8)	29.8 (21.4)	0.186
GGT [IU/L]	45 (42.3)	41.8 (50.1)	0.069	41.1 (42.5)	40.1 (65.6)	0.019
**Bone mineral density test results[b]**						
T-score >= -1.0	560 (44)	2316 (36.6)	0.151	1488 (41.6)	110 (40.9)	0.015
-2.5 < T-score < -1.0	509 (40)	2619 (41.4)	0.028	1456 (40.7)	112 (41.6)	0.018
T-score <= -2.5	204 (16)	1396 (22.1)	0.154	630 (17.6)	47 (17.5)	0.004

© 2023 Ko HY et al. *JAMA Network Open.*

Abbreviations: ASD, absolute standardized difference; AST, aspartate aminotransferase; ALT, alanine aminotransferase; DPP4i, dipeptidyl peptidase 4 inhibitors; GGT, gamma glutamyl transferase; GLP1RA, glucagon like peptide 1 receptor agonists; SD, standard deviation; SGLT2i, sodium glucose cotransporter 2 inhibitors; SGOT, serum glutamic oxaloacetic transaminase; SGPT, serum glutamic pyruvate transaminase.
[a]Laboratory test results were assessed within 3 years prior to the index date and the most recent value was included.
[b]Bone mineral density tests were conducted using DXA (dual-energy X-ray absorptiometry). The test results were assessed within 3 years prior to the index date and the most recent value was included.

© 2023 Ko HY et al. *JAMA Network Open.*

eTable 13. Association between the use of SGLT2 inhibitors and the risk of overall fractures among weighted populations with bone mineral density test results present.

Exposure	Patients[a]	Events	Person-years	Weighted IR[b] (95% CI)	Weighted HR (95% CI)
**SGLT2i vs. DPP4i**					
SGLT2 inhibitors	1,257	13	1,021	1.29 (0.68-2.20)	1.06 (0.58-1.96)
DPP4 inhibitors	6,103	79	5,785	1.22 (0.94-1.55)	1.00 [Reference]
**SGLT2i vs. GLP1RA**					
SGLT2 inhibitors	2,514	34	2,931	1.24 (0.81-1.81)	1.27 (0.30-5.49)
GLP-1 receptor agonists	250	2	169	0.67 (0.02-3.71)	1.00 [Reference]

Abbreviations: CI CI, confidence interval; DPP4i, dipeptidyl peptidase 4 inhibitors; GLP1RA, glucagon like peptide 1 receptor agonists; HR, hazard ratio; IR, incidence rate; SGLT2i, sodium glucose cotransporter 2 inhibitors

[a]Patients remained after trimming patients in the non-overlapping regions of the propensity score distributions.

[b]per 100 person-years

© 2023 Ko HY et al. *JAMA Network Open.*

eFigure 3. Cumulative incidence function plots for overall fractures among weighted populations

(A) SGLT2 inhibitors vs. DPP4 inhibitors

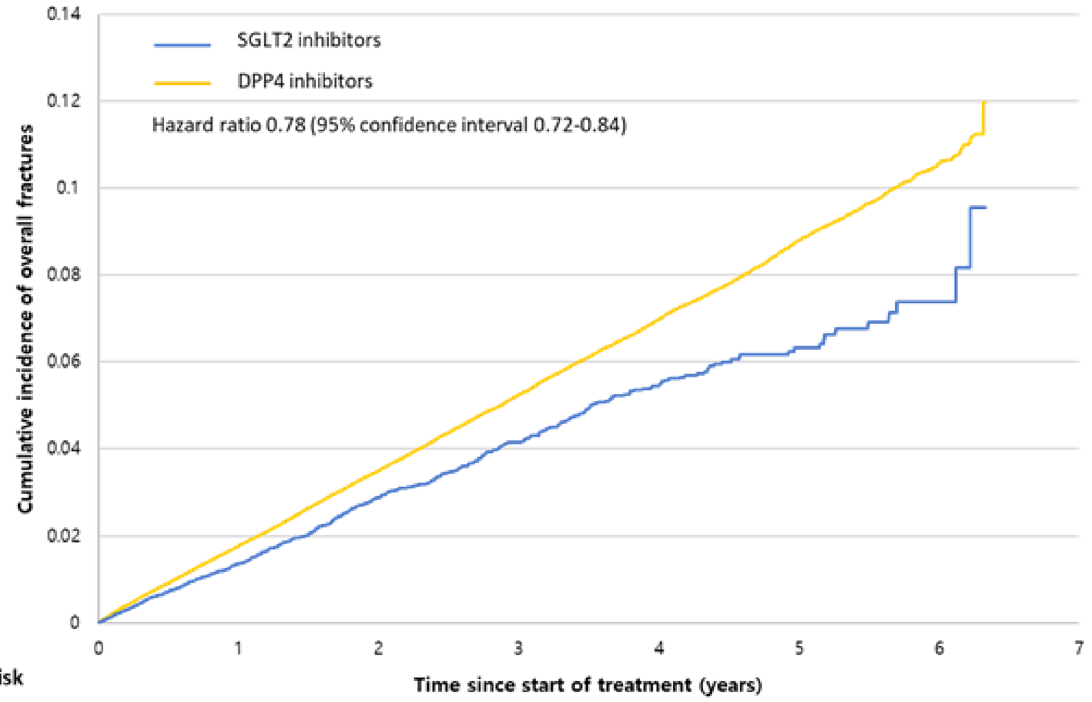

Number at risk								
SGLT2 inhibitor	37530	17786	10161	5982	2905	1120	194	0
DPP4 inhibitor	332004	186150	117680	75842	41184	17868	3876	0

(B) SGLT2 inhibitors vs. GLP-1 receptor agonists

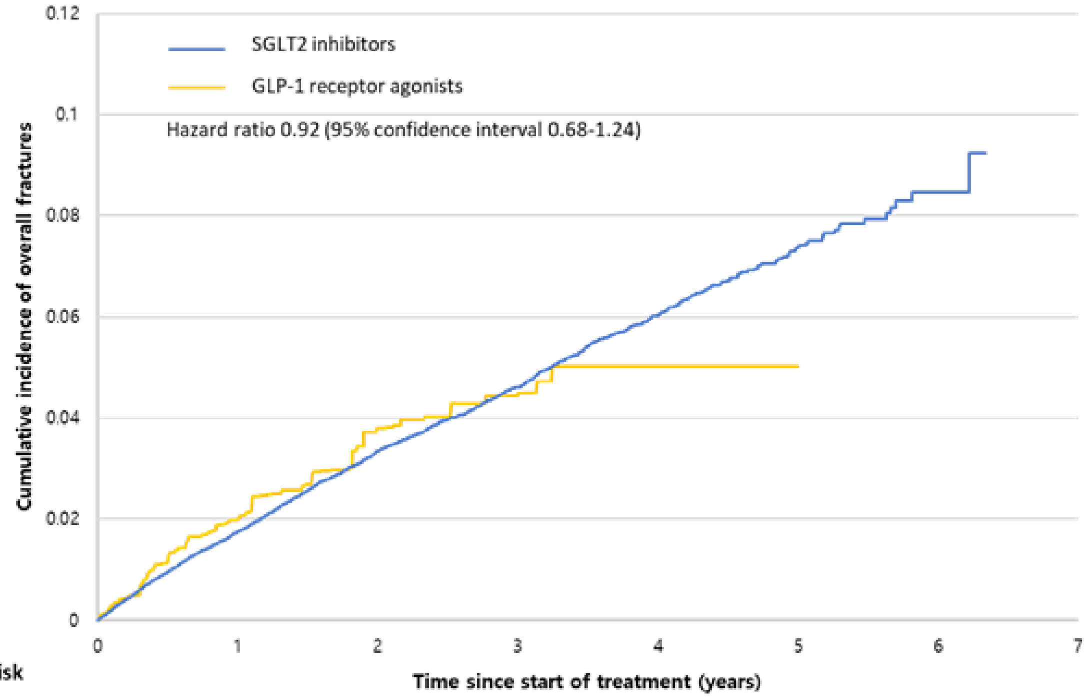

Number at risk								
SGLT2 inhibitor	111835	53158	28868	16339	7463	2296	309	0
GLP-1 receptor agonists	8177	1741	721	380	87	0	0	0

Abbreviations: DPP4, dipeptidyl peptidase 4; GLP-1, glucagon like peptide 1; SGLT2, sodium glucose cotransporter 2

© 2023 Ko HY et al. *JAMA Network Open.*

eFigure 4. Overall study design

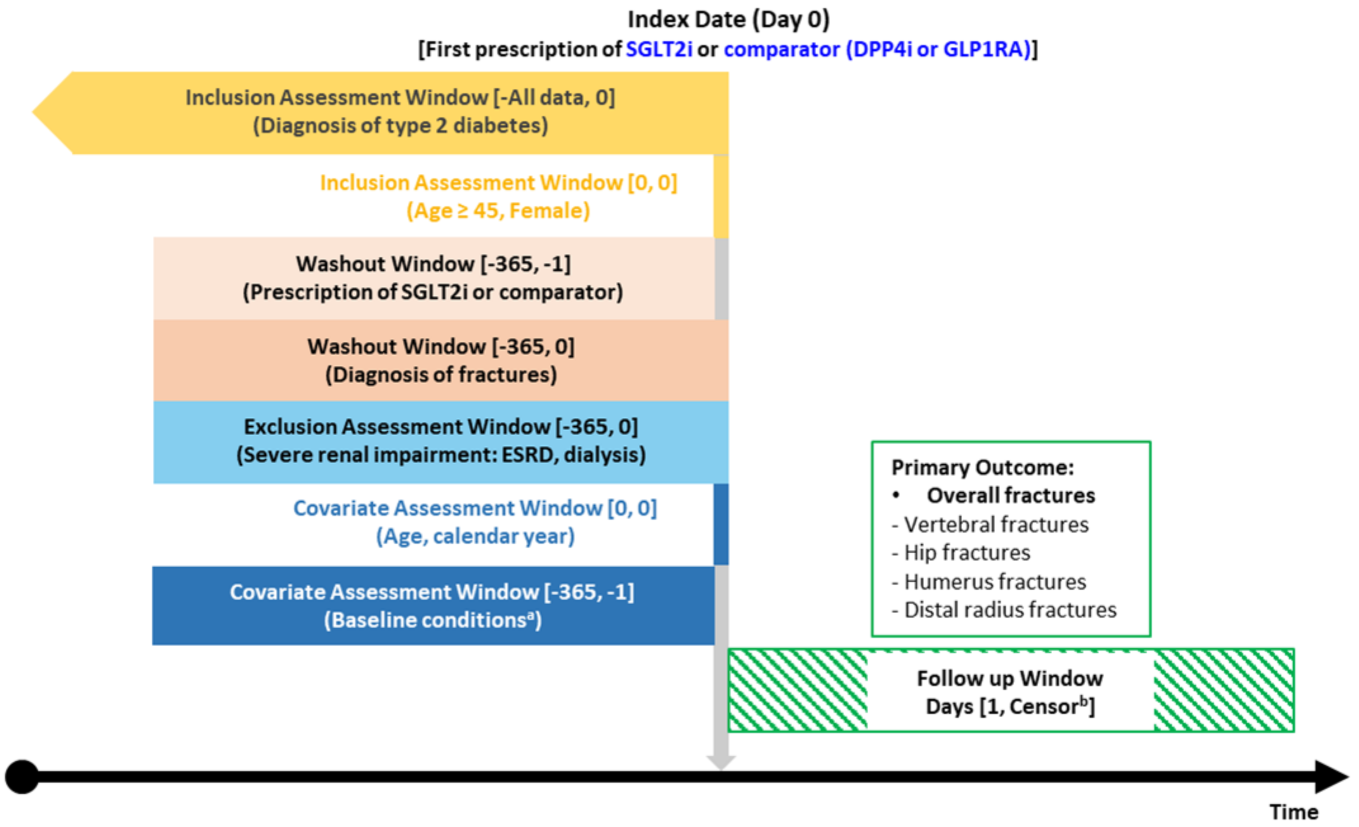

Abbreviations: DPP4i, dipeptidyl peptidase 4 inhibitor; ESRD, end-stage renal disease; GLP1RA, glucagon-like peptide 1 receptor agonist; SGLT2i, sodium glucose cotransporter 2 inhibitor

[a]Baseline conditions include diabetic related conditions, other comorbidities, fall related medications, diabetic medications, osteoporosis medications, other comedications, Charlson Comorbidity Index (CCI), proxies for diabetes and overall health status

[b]Earliest of the outcome of interest (fractures), switching or discontinuation of study drugs, death, end of the study period

© 2023 Ko HY et al. *JAMA Network Open.*

eFigure 5. Propensity score distribution of before and after weighting in both cohorts

	Before weighting	After weighting
**(A) SGLT2 inhibitors vs. DPP4 inhibitors** Post weighting c-statistic: 0.504	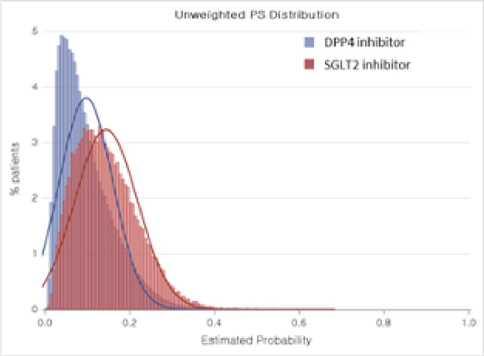	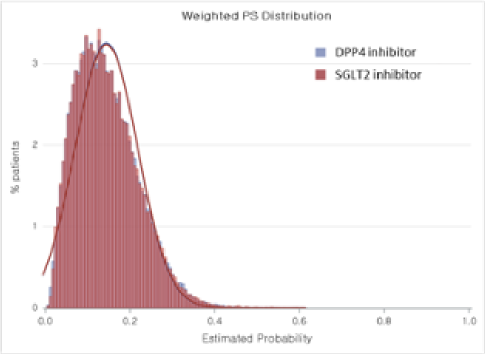
**(B) SGLT2 inhibitors vs. GLP-1 receptor agonists** Post weighting c-statistic: 0.567	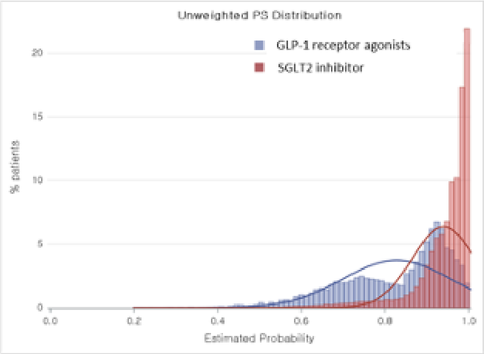	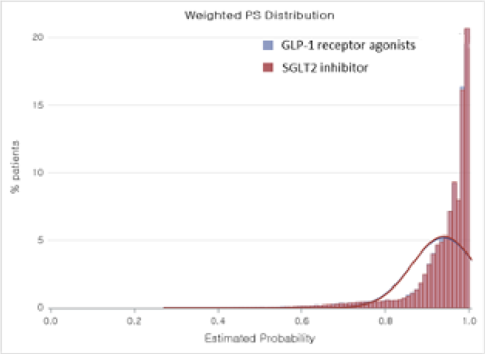

Abbreviations: DPP4, dipeptidyl peptidase 4; GLP-1, glucagon-like peptide 1; SGLT2, sodium glucose cotransporter 2

© 2023 Ko HY et al. *JAMA Network Open.*

eTable 14. International Classification of Diseases-10 (ICD-10) and domestic procedure codes for identification of fractures

Outcomes	Description	Codes
Vertebral fractures	Fracture of neck	S12
	Fracture of thoracic vertebra	S22.0
	Multiple fractures of thoracic spine	S22.1
	Fracture of lumbar vertebra	S32.0
	Multiple fractures of lumbar spine and pelvis	S32.7
	Fracture of spine, level unspecified	T08
	Fatigue fracture of vertebra	M48.4
	Collapsed vertebra, NEC	M48.5
	Collapsed vertebra in diseases classified elsewhere	M49.5
	Percutaneous vertebroplasty	N0471, N0472
	Percutaneous balloon kyphoplasty	N0473, N0474
	Closed reduction of fracture and/or dislocated spine	N0630
	Thoracic spine radiographic study	G430
	Thoracolumbar spine radiographic study	G440
	Lumbar spine radiographic study	G450
	Lumbosacral spine radiographic study	G460
Hip fractures	Fracture of neck of femur	S72.0
	Pertrochanteric fracture	S72.1
	Subtrochanteric fracture	S72.2
	Open reduction of fractured extremity (femur)	N0601, N0611
	Closed pinning for femur	N0991
	External fixation for pelvis or femur	N0981
	Closed reduction of fractured extremity (pelvis, femur)	N0641
	Bone traction	N0652
	Skin traction	N0654
	Total arthroplasty (hip)	N0711, N2070
	Hemiarthroplasty (hip)	N0715, N2710
Humerus fractures	Fracture of upper end of humerus	S42.2
	Fracture of shaft of humerus	S42.3
	Fracture of lower end of humerus	S42.4
	Open reduction with internal fixation for humerus or scapula	N0602, N0612
	Closed pinning for humerus or scapula	N0992
	Replacement hemiarthroplasty-shoulder	N2711, N2716
	Replacement total arthroplasty-shoulder	N2071, N2076
	Shoulder spica	T6010
	Velpeau cast	T6110
Distal radius fractures	Fracture of lower end of radius	S52.5
	Fracture of lower end of both ulna and radius	S52.6
	Open reduction with internal fixation for radius	N0607, N0617
	Open reduction with internal fixation for both radius and ulna	N0603, N0613
	Percutaneous pinning for radius	N0933
	Percutaneous pinning for radius and ulna	N0994
	External fixation for forearm bone	N0983
	Long-arm cast	T6020
	Short-arm cast	T6030

© 2023 Ko HY et al. *JAMA Network Open.*

Long-arm splint	T6151
Short-arm splint	T6152

eTable 15. International Classification of Diseases-10 (ICD-10) codes for identification of covariates

Covariates	Definitions[a]
**Diabetes related conditions**	
Diabetic nephropathy	E10.2: Type 1 diabetes mellitus with kidney complications<br>E11.2: Type 2 diabetes mellitus with kidney complications<br>E13.2: Other specified diabetes mellitus with kidney complications
Diabetic neuropathy	E10.4: Type 1 diabetes mellitus with neurological complications<br>E11.4: Type 2 diabetes mellitus with neurological complications<br>E13.4: Other specified diabetes mellitus with neurological complications<br>G53: Cranial nerve disorders in diseases classified elsewhere<br>G56: Mononeuropathies of upper limb<br>G57: Mononeuropathies of lower limb<br>G58: Other mononeuropathies<br>G59: Mononeuropathy in diseases classified elsewhere<br>G64: Other disorders of peripheral nervous system<br>G62.9: Polyneuropathy, unspecified<br>G90.0: Idiopathic peripheral autonomic neuropathy<br>G90.8: Other disorders of autonomic nervous system<br>G99.0: Autonomic neuropathy in diseases classified elsewhere
Diabetic retinopathy	E10.3: Type 1 diabetes mellitus with ophthalmic complications<br>E11.3: Type 2 diabetes mellitus with ophthalmic complications<br>E13.3: Other specified diabetes mellitus with ophthalmic<br>H33, H34, H35, H36, H54
Hypoglycemia	E16.0: Other disorders of pancreatic internal secretion<br>E16.1: Other hypoglycaemia<br>E16.2: Hypoglycaemia, unspecified<br>E11.63: Type 2 diabetes mellitus, with hypoglycemia<br>E13.63: Other specified diabetes mellitus, with hypoglycemia<br>E14.63: Unspecified diabetes mellitus, with hypoglycemia
**Charlson Comorbidity Index**	
Chronic pulmonary disease	ICD-10: I278, I279. J40, J41, J42, J43, J44, J45, J46, J47, J60, J61, J62, J63, J64, J65, J66, J67, J684, J701, J703
Rheumatic disease	ICD-10: M05, M06, M315, M32, M33, M34, M351, M353, M360
Diabetes with chronic complications	ICD-10: E102, E103, E104, E105, E107, E112, E113, E114, E115, E117, E122, E123, E124, E125, E127, E132, E133, E134, E135, E137, E142, E143, E144, E145, E147
Renal disease	ICD-10: I120, I131, N032, N033, N034, N035, N036, N037, N052, N053, N054, N055, N056, N057, N18, N19, N250, Z490, Z491, Z492, Z940, Z992
Congestive heart failure	ICD-10: I099, I110, I130, I132, I255, I420, I425, I426, I427, I428, I429, I43, I50, P290
Dementia	ICD-10: F00, F01, F02, F03, F051, G30, G311
Mild liver disease	ICD-10: B18, K700, K701, K702, K703, K709, K713, K714, K715, K717, K73, K74, K760, K762, K763, K764, K768, K769, Z944
Hemiplegia or paraplegia	ICD-10: G041, G114, G801, G802, G81, G82, G830, G831, G832, G833, G834, G839

© 2023 Ko HY et al. *JAMA Network Open.*

Any malignancy, including lymphoma and leukemia, except malignant neoplasm of skin	ICD-10: C00, C01, C02, C03, C04, C05, C06, C07, C08, C09, C10, C11, C12, C13, C14, C15, C16, C17, C18, C19, C20, C21, C22, C23, C24, C25, C26, C30, C31, C32, C33, C34, C37, C38, C39, C40, C41, C43, C45, C46, C47, C48, C49, C50, C51, C52, C53, C54, C55, C56, C57, C58, C60, C61, C62, C63, C64, C65, C66, C67, C68, C69, C70, C71, C72, C73, C74, C75, C76, C81, C82, C83, C84, C85, C88, C90, C91, C92, C93, C94, C95, C96, C97
Moderate or severe liver disease	ICD-10: I850, I859, I864, I982, K704, K711, K721, K729, K765, K766, K767
AIDS/HIV	ICD-10: B20, B21, B22, B24
Metastatic solid tumor	ICD-10: C77, C78, C79, C80

Other comorbidities

Covariates	Definitions[a]
Asthma	J45: Asthma<br>J46: Status asthmaticus
All other cancer	Any malignancy, including lymphoma and leukemia, metastatic solid tumor, except malignant neoplasm of skin and breast cancer<br>C00, C01, C02, C03, C04, C05, C06, C07, C08, C09, C10, C11, C12, C13, C14, C15, C16, C17, C18, C19, C20, C21, C22, C23, C24, C25, C26, C30, C31, C32, C33, C34, C37, C38, C39, C40, C41, C43, C45, C46, C47, C48, C49, C51, C52, C53, C54, C55, C56, C57, C58, C60, C61, C62, C63, C64, C65, C66, C67, C68, C69, C70, C71, C72, C73, C74, C75, C76, C77, C78, C79, C80, C81, C82, C83, C84, C85, C88, C90, C91, C92, C93, C94, C95, C96, C97
Breast cancer	C50: Malignant neoplasm of breast
Chronic kidney disease	N181: Chronic kidney disease, stage 1<br>N182: Chronic kidney disease, stage 2<br>N183: Chronic kidney disease, stage 3<br>N184: Chronic kidney disease, stage 4<br>N189: Chronic kidney disease, unspecified<br>N19: Unspecified kidney failure
Congestive heart failure	I50: Heart failure<br>I110: Hypertensive heart disease with (congestive) heart failure<br>I130: Hypertensive heart and renal disease with (congestive) heart failure<br>I132: Hypertensive heart and renal disease with both (congestive) heart failure and renal failure
COPD	J41: Simple and mucopurulent chronic bronchitis<br>J42: Unspecified chronic bronchitis<br>J43: Emphysema<br>J44: Other chronic obstructive pulmonary disease
Dementia	F01: Vascular dementia<br>F02: Dementia in other diseases classified elsewhere<br>F03: Unspecified dementia<br>G30: Alzheimer's disease<br>G310: Circumscribed brain atrophy<br>F00: Dementia in Alzheimer's disease<br>F051: Delirium superimposed on dementia<br>G311: Senile degeneration of brain, NEC
Epilepsy	G40: Epilepsy<br>G41: Status epilepticus
Gout	M10: Gout
Hyperlipidemia	E78: Disorders of lipoprotein metabolism and other lipidaemias
Hypertension	I10: Essential(primary) hypertension<br>I11: Hypertensive heart disease<br>I12: Hypertensive renal disease<br>I13: Hypertensive heart and renal disease<br>I15: Secondary hypertension

© 2023 Ko HY et al. *JAMA Network Open.*

Ischemic heart disease	I21: Acute myocardial infarction<br>I22: Subsequent myocardial infarction<br>I20: Angina pectoris<br>I25: Chronic ischaemic heart disease
Liver cirrhosis	K703: Alcoholic cirrhosis of liver<br>K717: Toxic liver disease with fibrosis and cirrhosis of liver<br>K74: Fibrosis and cirrhosis of liver
Osteoarthritis	M15: Polyarthrosis<br>M16: Coxarthrosis [arthrosis of hip]<br>M17: Gonarthrosis [arthrosis of knee]<br>M18: Arthrosis of first carpometacarpal joint<br>M19: Other arthrosis
Osteoporosis	M80: Osteoporosis with pathological fracture<br>M81: Osteoporosis without pathological fracture<br>M82: Osteoporosis in diseases classified elsewhere

**Covariates**	**Definitions**[a]
Parkinson's disease	G20: Parkinson's disease
Rheumatoid arthritis	M05: Seropositive rheumatoid arthritis<br>M06: Other rheumatoid arthritis
Stroke	I60: Subarachnoid haemorrhage<br>I61: Intracerebral haemorrhage<br>I62: Other nontraumatic intracranial haemorrhage<br>I63: Cerebral infarction<br>I64: Stroke, not specified as haemorrhage or infarction
Thyroid disease	E02: Subclinical iodine-deficiency hypothyroidism<br>E03: Other hypothyroidism<br>E05: Thyrotoxicosis<br>E06: Thyroiditis<br>E07: Other disorders of thyroid

[a]Comorbidities were ascertained from hospitalization or physician claims data (at least one inpatient or outpatient diagnosis in primary or secondary position) in a year prior to the index date.

© 2023 Ko HY et al. *JAMA Network Open.*

eTable 16. Anatomical Therapeutic Chemical (ATC) classification codes for identification of covariates

Covariates	Definitions[a]
**Fall-related medications**	
ACEi or ARBs	C09C, C09D, C09A, C09B
Anticholinergics	R06A
Benzodiazepines	N03AE, N05BA, N05CD
β-Blockers	C07
Calcium channel blockers	C08, C07FB, C09BB, C09DB
Diuretics	C03A, C07B, C07D, C03C, C03E, C03X, C07C, C08G
Nitrates	C01DA08
Opioids	N02A
Sedative hypnotics	N03AA, N05BE01, N05CA, N05CC01, N05CF
Tricyclic antidepressant	N06AA02, N06AA04, N06AA09, N06AA10, N06AA12, N06AA17, N06CA01
Antipsychotics	N05AA, N05AD, N05AE, N05AF, N05AG, N05AH, N05AL, N05AX
**Osteoporosis medications**	
Bisphosphonates	M05BA, M05BB
Calcium/vitamin D	A12AA04, A12AA13, A12AX
Parathyroid hormone/calcitonin	H05AA, H05BA
RANKL inhibitors	M05BX04
SERMs	G03XC
**Antihyperglycemic agents**	
SGLT2 inhibitors	A10BK
DPP4 inhibitors	A10BH
GLP-1 receptor agonists	A10BJ
α-Glucosidase inhibitors	A10BF
Insulin	A10A
Meglitinides	A10BX02, A10BX03, A10BX08
Metformin	A10BA02
Sulfonylureas	A10BB
Thiazolidinediones	A10BG
Combinations	A10BD
**Other comedications**	
Anticoagulants	B01AA, B01AB01, B01AB04, B01AB05, B01AB06, B01AB07, B01AB08, B01AB012, B10AF01, B10AF02, B10AF03, B01AX05
Anticonvulsants	N02BF, N03AB02, N03AB03, N03AB05, N03AB52, N03AD01, N03AD51, N03AF, N03AG01, N03AG04, N03AX
Antidepressants	N06AB, N06AX11, N06AX16, N06AX21, N06AX23
Aromatase inhibitors	L02BG03, L02BG06, L02BG04
Oral corticosteroids	H02AA02, H02AB, H02BX
Hormone replacement therapy	G03CA57, G03CX, G03DB01, G03DB08, G03F, G03H, G03XA01
Immunosuppressants	L04A
NSAIDs	M01AB05, M01AB08, M01AB15, M01AB16, M01AC01, M01AC06, M01AC56, M01AE01, M01AE02, M01AE03, M01AE04, M01AE09, M01AE13, M01AE14, M01AE17, M01AH01, M01AH05, M01AH07
Platelet inhibitors	B01AC, B01AD01, B01AD04, B01AD11
Proton-pump inhibitors	A02BC
Statins	C01AA

Abbreviations: ACE, angiotensin converting enzyme; ARBs, angiotensin receptor blockers; DPP4i, dipeptidyl peptidase 4 inhibitors; GLP1RA, glucagon like peptide 1 receptor agonists; NSAIDs, nonsteroidal anti-inflammatory drugs; RANKL, receptor activator of nuclear factors kappa B ligand; SERMs, selective estrogen receptor modulator; SGLT2i, sodium glucose cotransporter 2 inhibitors
[a]Comedications were assessed in a year prior to the index date.

© 2023 Ko HY et al. *JAMA Network Open.*
