# Supplementary material for: Sodium-Glucose Cotransporter 2 Inhibitors vs Incretin-Based Drugs and Risk of Fractures for Type 2 Diabetes
Source: JAMA Netw Open. 2023 Sep 26;6(9):e2335797. doi: 10.1001/jamanetworkopen.2023.35797 (PMC10523172; doi:10.1001/jamanetworkopen.2023.35797)
Supplement: Supplement 2. — Data Sharing Statement [file jamanetwopen-e2335797-s002.pdf]

Data Sharing Statement

Ko. Sodium-Glucose Cotransporter 2 Inhibitors vs Incretin-Based Drugs and Risk Of Fractures For Type 2 Diabetes. *JAMA Netw Open*. Published September 26, 2023. doi:10.1001/jamanetworkopen.2023.35797

Data
Data available: No
